# Vagus nerve stimulation primes platelets and reduces bleeding in hemophilia A male mice

**DOI:** 10.1038/s41467-023-38505-6

**Published:** 2023-06-01

**Authors:** Carlos E. Bravo-Iñiguez, Jason R. Fritz, Shilpa Shukla, Susmita Sarangi, Dane A. Thompson, Seema G. Amin, Tea Tsaava, Saher Chaudhry, Sara P. Valentino, Hannah B. Hoffman, Catherine W. Imossi, Meghan E. Addorisio, Sergio I. Valdes-Ferrer, Sangeeta S. Chavan, Lionel Blanc, Christopher J. Czura, Kevin J. Tracey, Jared M. Huston

**Affiliations:** 1 Institute of Bioelectronic Medicine, The Feinstein Institutes for Medical Research at Northwell Health, 350 Community Drive, Manhasset, NY 11030 USA; 2 Elmezzi Graduate School of Molecular Medicine at Northwell Health, 350 Community Drive, Manhasset, NY 11030 USA; 3 Department of Pediatric Hematology and Oncology, Cohen Children’s Medical Center, Northwell Health, Lake Success, NY 11040 USA; 4 Department of Surgery, Northwell Health, 300 Community Drive, Manhasset, NY 11030 USA; 5 Institute of Molecular Medicine, The Feinstein Institutes for Medical Research at Northwell Health, 350 Community Drive, Manhasset, NY 11030 USA; 6 Center for Autoimmune, Musculoskeletal and Hematopoietic Diseases, The Feinstein Institutes for Medical Research at Northwell Health, 350 Community Drive, Manhasset, NY 11030 USA; 7 Departments of Molecular Medicine and Pediatrics, Donald and Barbara Zucker School of Medicine at Hofstra/Northwell, 500 Hofstra Boulevard, Hempstead, NY 11549 USA; 8 Department of Science Education, Donald and Barbara Zucker School of Medicine at Hofstra/Northwell, 500 Hofstra Boulevard, Hempstead, NY 11549 USA

**Keywords:** Haematological diseases, Calcium signalling, Coagulation system, Autonomic nervous system, Neuroimmunology

## Abstract

Deficiency of coagulation factor VIII in hemophilia A disrupts clotting and prolongs bleeding. While the current mainstay of therapy is infusion of factor VIII concentrates, inhibitor antibodies often render these ineffective. Because preclinical evidence shows electrical vagus nerve stimulation accelerates clotting to reduce hemorrhage without precipitating systemic thrombosis, we reasoned it might reduce bleeding in hemophilia A. Using two different male murine hemorrhage and thrombosis models, we show vagus nerve stimulation bypasses the factor VIII deficiency of hemophilia A to decrease bleeding and accelerate clotting. Vagus nerve stimulation targets acetylcholine-producing T lymphocytes in spleen and α7 nicotinic acetylcholine receptors (α7nAChR) on platelets to increase calcium uptake and enhance alpha granule release. Splenectomy or genetic deletion of T cells or α7nAChR abolishes vagal control of platelet activation, thrombus formation, and bleeding in male mice. Vagus nerve stimulation warrants clinical study as a therapy for coagulation disorders and surgical or traumatic bleeding.

## Abstract (web summary)

Coagulation factor VIII deficiency in hemophilia A disrupts clotting and prolongs bleeding. Here, the authors show that vagus nerve stimulation bypasses this defect and improves hemostasis in hemophilia A mice through a mechanism requiring acetylcholine-secreting ChAT^+^ T lymphocytes in spleen and α7nAChR on circulating platelets.

## Introduction

Primary hemostasis after blood vessel injury induces local vasoconstriction and recruitment of circulating platelets to form an initial platelet plug^1^. Secondary hemostasis occurs on the surface of activated platelets where the tissue factor and contact activation pathways generate thrombin (coagulation factor II), which cross-links soluble fibrin into insoluble fibrin to stabilize developing clot that stops bleeding^2,3^. Hemophilia A results from congenital or acquired impairment in coagulation factor VIII activity, most often via genetic mutations that reduce or eliminate factor VIII production^4^. Without essential factor VIII, the contact activation pathway cannot help generate sufficient thrombin and insoluble fibrin, resulting in clot instability. Clinical hallmarks of hemophilia A include prolonged hemorrhage after injury and spontaneous bleeding into muscles and joints. Because the incidence of bleeding complications correlates with circulating factor VIII levels, traditional and current therapies focus on replacing factor VIII through exogenous infusions^5,6^. Administration of recombinant factor VIII concentrate mitigates transmission of hepatitis or human immunodeficiency virus^7^. However, more than 30% of children with severe hemophilia A cease responding to therapy because they develop neutralizing (inhibitor) factor VIII antibodies^8–10^. Treatment then shifts to prothrombin complex concentrates or recombinant factor VIIa to bypass factor VIII, but these alternative thrombotic agents are very expensive and associated with significant morbidity and mortality^11,12^.

The autonomic nervous system regulates tissue perfusion and hemostasis^13,14^. Norepinephrine release from perivascular sympathetic neurons mediates arteriolar vasoconstriction to decrease blood flow and facilitate clot formation after injury^15^. Preganglionic sympathetic neurons stimulate the adrenal medulla to secrete epinephrine that activates circulating platelets, the body’s primary effector cells that support clotting^16,17^. Parasympathetic activation mediated by the vagus nerve can support clot formation following traumatic hemorrhage by slowing heart rate to reduce blood pressure and tissue perfusion^18,19^. Preclinical evidence shows that electrical stimulation of the cervical vagus nerve significantly reduces blood loss and duration of bleeding after soft tissue injury in swine^20^. Vagus nerve stimulation accelerates clot initiation as measured by rotational thromboelastography (RoTEG), a viscoelastic technique to quantify interactions of plasma coagulation factors and inhibitors with blood cells^20^. Vagus nerve stimulation increases thrombin generation at the injury site without significantly changing circulating thrombin levels^20^. Accordingly, here we reasoned vagus nerve stimulation might enhance clot formation and reduce bleeding in hemophilia A mice.

## Results

### Vagus nerve stimulation reduces bleeding in hemophilia mice

**Fig. 1:**
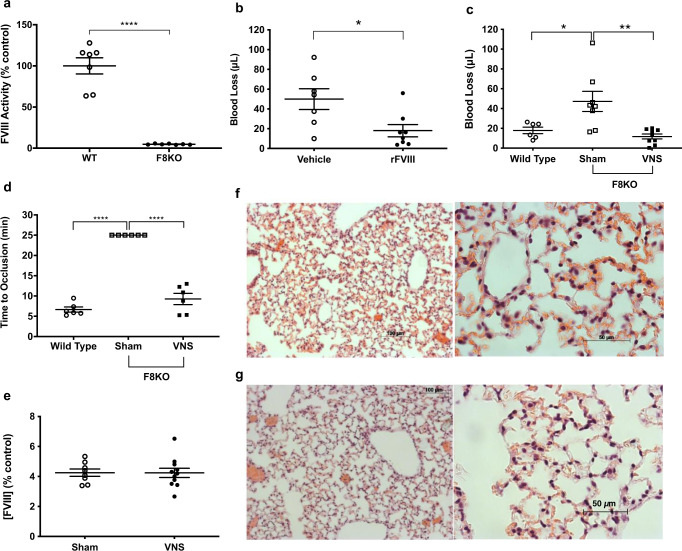
Vagus nerve stimulation reduces traumatic blood loss and accelerates clot formation in hemophilia A mice. **a** Circulating factor VIII activity in wild-type and factor VIII deficient (F8KO) hemophilia A mice. Data were presented as mean ± s.e.m. Sham (*n* = 7) and VNS (*n* = 7) mice per group. ****p < 0.0001 vs. control. Statistical significance was determined by unpaired two-tailed Student’s *t*-test. The figure represents pooled results from two experiments performed independently. **b** Hemophilia A (F8KO) mice received rFVIII (Advate®, 200 U/kg, r.o.) or vehicle before tail transection. Data were presented as mean ± s.e.m. Sham (*n* = 7) and VNS (*n* = 8) mice per group. *p < 0.018 vs. vehicle. Statistical significance was determined by unpaired two-tailed Student’s *t*-test. The figure represents pooled results from three experiments performed independently. **c** Hemophilia A (F8KO) mice received vagus nerve stimulation or sham stimulation before tail transection. Data were presented as mean ± s.e.m. WT (*n* = 6), Sham (*n* = 8), and VNS (*n* = 9) mice per group. *p < 0.05 vs. wild type. **p < 0.01 vs. sham. Statistical significance was determined by one-way ANOVA (Tukey). The figure represents pooled results from three experiments performed independently. **d** Hemophilia A (F8KO) mice received vagus nerve stimulation or sham stimulation before carotid artery injury. Data were presented as mean ± s.e.m. WT (*n* = 6), Sham (*n* = 6), and VNS (*n* = 6) mice per group. ****p < 0.0001 vs. wild type. ****p < 0.0001 vs. sham. Statistical significance was determined by one-way ANOVA (Tukey). The figure represents pooled results from three experiments performed independently. **e** Hemophilia A (F8KO) mice received vagus nerve stimulation or sham stimulation before blood collection to determine factor VIII activity. Data were presented as mean ± s.e.m. Sham (*n* = 8) and VNS (*n* = 11) mice per group. *p* = 0.97 vs. sham. Statistical significance was determined by unpaired two-tailed Student’s *t*-test. The figure represents pooled results from two experiments performed independently. **f** Representative images of lungs from hemophilia A (F8KO) mice after sham stimulation and tail transection. Experiments were performed independently at least three times with similar results. **g** Representative images of lungs from hemophilia A (F8KO) mice after vagus nerve stimulation and tail transection. Experiments were performed independently at least three times with similar results. Source data are provided as a Source Data File.

To study vagus nerve stimulation in hemophilia A, we utilized factor VIII-knockout (F8KO) mice derived via targeted mutation of the factor VIII gene resulting in defective protein production^21^. To validate the bleeding model, we measured circulating factor VIII activity in F8KO mice and found it is significantly reduced compared with wild-type control animals (Fig. 1a). We then administered recombinant factor VIII concentrate, a clinical hemophilia A therapy, to F8KO mice before tail transection causing uncontrolled arterial hemorrhage, and recorded a significant 64% reduction in blood loss as compared with vehicle treatment (Fig. 1b). In comparison to factor VIII treatment, administering five minutes of electrical vagus nerve stimulation to F8KO mice significantly reduces blood loss by 75% as compared with sham stimulation (Fig. 1c). To determine if vagus nerve stimulation accelerates clot formation in F8KO mice, we utilized a ferric chloride-induced, carotid artery endothelial injury model that quantifies platelet activation and thrombus deposition^22,23^. Compared with wild-type controls, we observed significantly longer times to carotid occlusion in F8KO mice (Fig. 1d). In fact, none of the F8KO mice achieved total vessel occlusion after injury (Fig. 1d). Following vagus nerve stimulation, however, time to carotid occlusion in F8KO mice is significantly reduced and similar to wild-type animals (Fig. 1d). Because the vagus nerve innervates liver which produces factor VIII, we wondered if vagus nerve stimulation increases factor VIII synthesis to normalize clot formation in F8KO mice^24^. However, our results show vagus nerve stimulation does not increase systemic factor VIII activity as compared with sham stimulation (Fig. 1e). We then analyzed lung sections for signs of systemic hypercoagulability after tail injury. Compared with sham stimulation, mice that received vagus nerve stimulation demonstrated normal tissue architecture without evidence of arterial clots, alveolar congestion, or hemorrhage associated with thromboembolism (Fig. 1f, g). Together these findings show vagus nerve stimulation reduces blood loss and accelerates local clot formation in hemophilia A mice without increasing systemic factor VIII activity or inducing pulmonary thrombosis.

### Vagus nerve stimulation improves hemostasis via the spleen

**Fig. 2:**
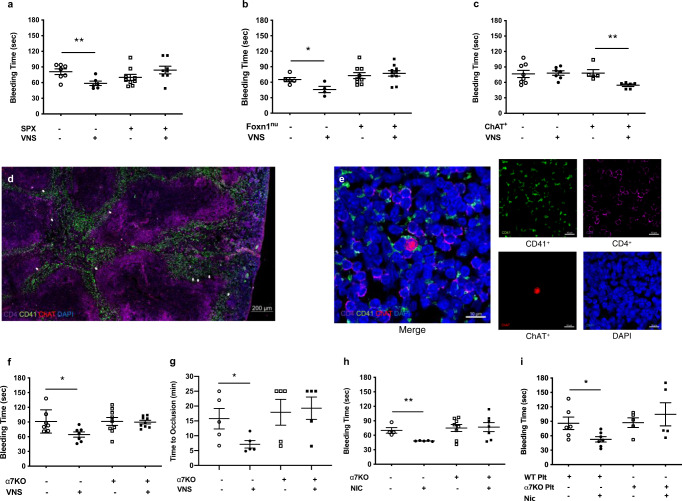
Vagus nerve stimulation harnesses choline acetyltransferase-expressing T lymphocytes in the spleen to stimulate circulating platelets via α7nAChR. **a** C57BL6/J mice underwent splenectomy or sham-splenectomy followed by vagus nerve stimulation or sham stimulation before tail transection. Data were presented as mean ± s.e.m. Sham SPX + Sham VNS (*n* = 7), Sham SPX + VNS (*n* = 6), SPX + Sham VNS (*n* = 9), SPX + VNS (*n* = 8) mice per group. **p = 0.009 vs. sham in sham-splenectomy group. *p* = 0.15 VNS vs. sham after SPX. Statistical significance was determined by unpaired two-tailed Student’s *t*-test. The figure represents pooled results from two experiments performed independently. **b** Wild-type BALB/c or T lymphocyte deficient (Foxn1^nu^) mice received vagus nerve stimulation or sham stimulation before tail transection. Data were presented as mean ± s.e.m. WT + Sham VNS (*n* = 5), WT + VNS (*n* = 4), Foxn1^nu^ + Sham VNS (*n* = 9), Foxn1^nu^ + VNS (*n* = 10) mice per group. *p = 0.03 vs. sham in WT mice. *p* = 0.60 VNS vs. sham in Foxn1^nu^ mice. Statistical significance was determined by unpaired two-tailed Student’s *t*-test. The figure represents pooled results from two experiments performed independently. **c** T lymphocyte deficient (Foxn1^nu^) mice were reconstituted with ChAT-eGFP^+^ or ChAT-eGFP^-^ cells followed by vagus nerve stimulation or sham stimulation before tail transection. Data were presented as mean ± s.e.m. ChAT-eGFP^−^ + Sham VNS (*n* = 8), ChAT-eGFP^−^ + VNS (*n* = 7), ChAT-eGFP^+^ + Sham VNS (*n* = 5), ChAT-eGFP^+^ + VNS (*n* = 6) mice per group. **p = 0.005 vs. sham in ChAT-eGFP^+^ cells. *p* = 0.85 VNS vs. sham in ChAT-eGFP^-^ cells. Statistical significance was determined by unpaired two-tailed Student’s *t*-test. The figure represents pooled results from two experiments performed independently. **d** Representative confocal microscopy image (10X) of spleen from ChAT-TdTomato mouse with immunostaining of CD41^+^ platelets (green), CD4^+^ T lymphocytes (purple), and ChAT-eGFP^+^ cells (red) throughout spleen but mostly in the peripheral white pulp (arrows). Scale bar = 200 µm. Experiments were performed independently at least three times with similar results. **e** Representative merged confocal microscopy image (left, 63X) of spleen from ChAT-TdTomato mouse showing CD4^+^ ChAT-eGFP^+^ T lymphocyte (red and pink, center) in direct contact with CD41^+^ platelets (green). Scale bar = 10 µm. Representative confocal microscopy of individual color channels (right, 63X) including CD41^+^ platelets (green), CD4^+^ T lymphocytes (purple), ChAT-eGFP^+^ cell (red), and DAPI (blue). Scale bars = 10 µm. Experiments were performed independently at least three times with similar results. **f** Wild-type or α7nAChR-deficient (α7KO) mice received vagus nerve stimulation or sham stimulation before tail transection. Data were presented as mean ± s.e.m. WT + Sham VNS (*n* = 7), WT + VNS (*n* = 7), α7KO + Sham VNS (*n* = 9), α7KO + VNS (*n* = 9) mice per group. *p = 0.025 vs. sham in WT mice. *p* = 0.87 VNS vs. sham in α7KO mice. Statistical significance was determined by unpaired two-tailed Student’s *t*-test. The figure represents pooled results from two experiments performed independently. **g** Wild-type or α7nAChR-deficient (α7KO) mice received vagus nerve stimulation or sham stimulation before carotid artery injury. Data were presented as mean ± s.e.m. WT + Sham VNS (*n* = 5), WT + VNS (*n* = 5), α7KO + Sham VNS (*n* = 5), α7KO + VNS (*n* = 5) mice per group. *p = 0.046 vs. sham in WT mice. *p* = 0.82 VNS vs. sham in α7KO mice. Statistical significance was determined by unpaired two-tailed Student’s *t*-test. The figure represents pooled results from two experiments performed independently. **h** Wild-type or α7nAChR-deficient (α7KO) mice received nicotine or vehicle before tail transection. Data were presented as mean ± s.e.m. WT + Vehicle (*n* = 4), WT + Nicotine (*n* = 5), α7KO + Vehicle (*n* = 8), α7KO + Nicotine (*n* = 7). **p = 0.0037 vs. vehicle in WT mice. *p* = 0.88 Nic vs. vehicle in α7KO mice. Statistical significance was determined by unpaired two-tailed Student’s *t*-test. The figure represents pooled results from two experiments performed independently. **i** α7nAChR-deficient (α7KO) mice were reconstituted with platelets from wild-type or α7KO mice, followed by treatment with nicotine or vehicle before tail transection. Data are presented as mean ± s.e.m. WT platelets + Vehicle (*n* = 6), WT Platelets + Nicotine (*n* = 7), α7KO Platelets + Vehicle (*n* = 5), α7KO Platelets + Nicotine (*n* = 5). *p = 0.029 vs. vehicle with WT platelets. *p* = 0.52 Nic vs. vehicle with α7KO platelets. Statistical significance was determined by unpaired two-tailed Student’s *t*-test. The figure represents pooled results from two experiments performed independently. Source data are provided as a Source Data File.

Our previous studies demonstrate that vagus nerve stimulation inhibits circulating neutrophil migration to sites of peripheral inflammation and decreases expression of CD11b, a beta(2)-integrin important to cellular adhesion and chemotaxis, but only in the presence of an intact spleen^25,26^. Accordingly, we reasoned that vagus nerve stimulation modulates peripheral hemostasis by targeting circulating platelets in the spleen. Next, we performed splenectomy or sham surgery before tail transection. We did not observe a difference in bleeding times between splenectomized and sham-splenectomized animals (Fig. 2a). Vagus nerve stimulation significantly decreases bleeding time in mice after sham-splenectomy, but fails to control bleeding in splenectomized animals (Fig. 2a).

### Splenic ChAT-EGFP^+^ T lymphocytes control hemostasis

Prior evidence establishes that vagus nerve stimulation induces a distinct subset of splenic memory T cells (CD4^+^ CD44^high^ CD62^low^ ChAT-EGFP^+^) expressing choline acetyltransferase, the enzyme that catalyzes acetylcholine synthesis, to increase local acetylcholine levels and inhibit cytokine production by tissue macrophages^27^. To determine the role of splenic ChAT-EGFP^+^ T lymphocytes in hemostasis, we first performed tail transections in athymic nude (Foxn1^nu^) mice lacking mature functional T lymphocytes. We did not observe a difference in bleeding times between Foxn1^nu^ mice and wild-type BALC/c mice (Fig. 2b). Vagus nerve stimulation significantly reduces bleeding time in wild-type mice as compared with sham stimulation, but fails to decrease bleeding time in Foxn1^nu^ mice (Fig. 2b). We then isolated ChAT-EGFP^+^ or control ChAT-EGFP^-^ splenic cells from ChAT(BAC)-EGFP mice and transferred them into T cell-deficient Foxn1^nu^ mice before the tail injury. Vagus nerve stimulation significantly reduces bleeding time in Foxn1^nu^ mice reconstituted with ChAT-EGFP^+^ T lymphocytes, but fails to shorten the bleeding time in animals receiving ChAT-EGFP^-^ T cells (Fig. 2c). Collectively, these findings suggest vagus nerve stimulation reduces bleeding through a mechanism that requires acetylcholine-secreting ChAT-EGFP^+^ T lymphocytes in spleen.

### Splenic ChAT-EGFP^+^ T lymphocytes contact systemic platelets

Acetylcholine and other cholinergic agonists enhance human platelet activation via α7 nicotinic acetylcholine receptors (α7nAChR)^28^. Considering one-third of body platelets reside as an exchangeable circulating pool in the spleen^29,30^, we reasoned that splenic ChAT-EGFP^+^ T cell acetylcholine secretion controls systemic platelet function. To explore this relationship, we performed immunohistochemistry on spleens from ChAT-tdTomato mice that express the red fluorescent tdTomato protein in cholinergic cells. White pulp tissue in the spleen consists of centrally-located periarteriolar lymphoid sheaths (PALSs) containing mostly T lymphocytes, with B lymphocyte follicles located peripherally^31,32^. Red pulp tissue surrounds white pulp and contains arterioles, capillaries, sinuses, and venules in an open circulation with circulating blood cells^31,32^. The marginal zone, located at the interface between white and red pulp, contains lymphocytes and circulating cells^32^. In agreement, we observed CD4^+^ T lymphocytes in direct contact with CD41^+^ stained platelets in this region (Fig. 2d). We observed ChAT expression throughout the spleen but mostly in the peripheral white pulp (Fig. 2d), which is consistent with previous work showing a higher frequency of ChAT^+^ T and B lymphocytes in this region^33^. Furthermore, we observed CD4^+^ ChAT-EGFP^+^ T lymphocytes in direct contact with CD41^+^ stained platelets (Fig. 2e), thus providing an anatomical basis for T lymphocyte modulation of platelet function via acetylcholine secretion.

### Vagus nerve stimulation controls bleeding via α7nAChR

Next, we studied tail hemorrhage in α7nAChR-deficient (α7KO) mice. We did not observe a difference in bleeding time between α7KO and wild-type littermate mice (Fig. 2f). Vagus nerve stimulation significantly reduces tail bleeding time in wild-type mice as compared with sham stimulation, but fails to control bleeding in α7KO mice (Fig. 2f). We then studied clot formation following carotid injury in α7KO mice. There is no difference in vascular occlusion time between α7KO and wild-type mice (Fig. 2g). Vagus nerve stimulation significantly reduces occlusion time in wild-type animals as compared with sham stimulation, but fails to decrease occlusion time in α7KO mice (Fig. 2g). Together these findings indicate vagus nerve stimulation accelerates thrombosis and decreases bleeding through a mechanism requiring α7nAChR.

### Platelet α7nAChR is sufficient to control hemostasis

To confirm platelet α7nAChR modulates hemostasis, we first administered the pharmacological α7nAChR agonist nicotine to mice before the tail injury. Similar to vagus nerve stimulation, nicotine significantly decreases bleeding time in wild-type mice as compared with vehicle treatment, but fails to reduce bleeding time in α7KO mice (Fig. 2h). To restrict α7nAChR expression to platelets, we transferred platelets from wild-type or α7nAChR-deficient donor mice into α7KO animals before tail transection. Nicotine significantly reduces bleeding time in α7KO mice reconstituted with wild-type platelets, but not in animals given platelets from α7nAChR-deficient donors (Fig. 2i), indicating that platelet-specific α7nAChR is sufficient to control hemostasis. To explore platelet α7nAChR function, we initially measured receptor expression and observed that similar percentages of platelets express α7nAChR after vagus nerve stimulation as compared to sham stimulation (Supplementary Fig. 1a). Moreover, on α7nAChR-positive platelets, we did not observe a difference in receptor expression level after vagus nerve stimulation as compared with sham stimulation (Supplementary Fig. 1b).

### α7nAChR increases platelet cytosolic calcium

**Fig. 3:**
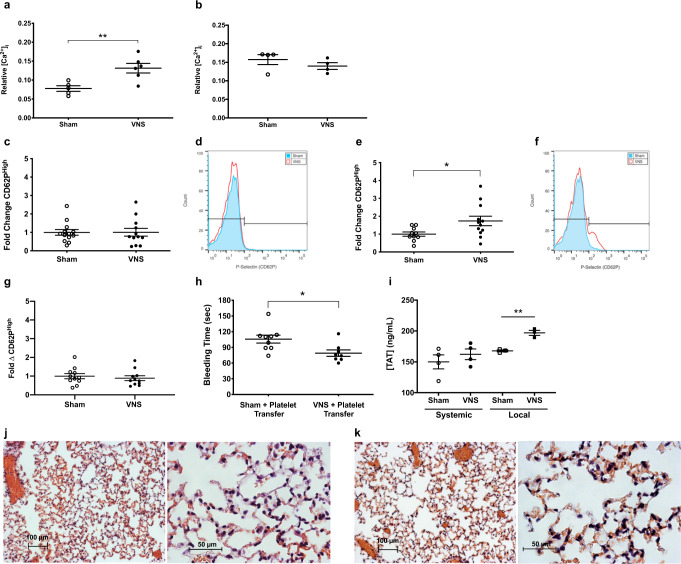
Vagus nerve stimulation requires α7nAChR to increase platelet cytosolic calcium, enhance cellular activation, and accelerate local clot formation to reduce bleeding. **a** C57BL6/J mice received vagus nerve stimulation or sham stimulation before harvesting circulating platelets to measure basal cytosolic calcium uptake. Data were presented as mean ± s.e.m. Sham (*n* = 5) and VNS (*n* = 6) mice per group. **p = 0.0073 vs. sham. Statistical significance was determined by unpaired two-tailed Student’s *t*-test. The figure represents pooled results from two experiments performed independently. **b** α7nAChR-deficient (α7KO) mice received vagus nerve stimulation or sham stimulation before harvesting circulating platelets to measure basal cytosolic calcium uptake. Data were presented as mean ± s.e.m. Sham (*n* = 4) and VNS (*n* = 4) mice per group. *p* = 0.32 vs. sham. Statistical significance was determined by unpaired two-tailed Student’s *t*-test. The figure represents pooled results from two experiments performed independently. **c** C57BL6/J mice received vagus nerve stimulation or sham stimulation before harvesting platelets for analysis of P-selectin (CD62P) expression. Data were presented as mean ± s.e.m. Sham (*n* = 13) and VNS (*n* = 12) mice per group. *p* = 0.98 vs. sham. Statistical significance was determined by unpaired two-tailed Student’s *t*-test. The figure represents pooled results from three experiments performed independently. **d** Representative FACS histogram of platelet P-selectin (CD62P) expression after vagus nerve stimulation or sham stimulation. Experiments were performed independently three times with similar results. **e** C57BL6/J mice received vagus nerve stimulation or sham stimulation before harvesting platelets, ex vivo thrombin stimulation, and analysis of P-selectin (CD62P) expression. Data were presented as mean ± s.e.m. Sham (*n* = 10) and VNS (*n* = 12) mice per group. *p = 0.029 vs. sham. Statistical significance was determined by unpaired two-tailed Student’s *t*-test. The figure represents pooled results from three experiments performed independently. **f** Representative FACS histogram of platelet P-selectin (CD62P) expression after vagus nerve stimulation or sham stimulation followed by ex vivo thrombin stimulation. Experiments were performed independently three times with similar results. **g** α7nAChR-deficient (α7KO) mice received vagus nerve stimulation or sham stimulation before harvesting platelets, ex vivo thrombin stimulation, and analysis of P-selectin (CD62P) expression. Data were presented as mean ± s.e.m. Sham (*n* = 11) and VNS (*n* = 11) mice per group. *p* = 0.57 vs. sham. Statistical significance was determined by unpaired two-tailed Student’s *t*-test. The figure represents pooled results from three experiments performed independently. **h** Platelets from uninjured C57BL6/J mice receiving vagus nerve stimulation or sham stimulation were transferred into naïve animals before tail transection. Data were presented as mean ± s.e.m. Sham (*n* = 9) and VNS (*n* = 8) mice per group. *p = 0.016 vs. sham. Statistical significance was determined by unpaired two-tailed Student’s *t*-test. The figure represents pooled results from two experiments performed independently. **i** C57BL6/J mice received vagus nerve stimulation or sham stimulation before tail transection and collection of systemic or local shed blood for measurement of (TAT) complexes. Data were presented as mean ± s.e.m. Sham VNS + Systemic (*n*-4), VNS + Systemic (*n* = 4), Sham VNS + Local (*n* = 3), VNS + Local (*n* = 3) mice per group. **p = 0.003 vs. sham in local blood. *p* = 0.42 VNS vs. sham in systemic blood. Statistical significance was determined by unpaired two-tailed Student’s *t*-test. The figure represents pooled results from two experiments performed independently. **j** Representative low power (left, scale bar = 100 µm) and high power (right, scale bar = 50 µm) lung images from C57BL6/J mice after sham stimulation and tail transection. Experiments were performed independently three times with similar results. **k** Representative low power (left, scale bar = 100 µm) and high power (right, scale bar = 50 µm) lung images from C57BL6/J mice after vagus nerve stimulation and tail transection. Experiments were performed independently three times with similar results. Source data are provided as a Source Data File.

Previous in vitro experiments demonstrate that α7nAChR modulates human platelet calcium uptake^28^, and intracellular calcium governs essential platelet activation pathways^34,35^. By quantifying platelet cytosolic calcium levels, we found vagus nerve stimulation significantly increases basal intracellular calcium concentrations as compared with sham stimulation (Fig. 3a). Vagus nerve stimulation fails to increase platelet cytosolic calcium in α7nAChR-deficient mice (Fig. 3b), or when the extracellular calcium ion source is removed (Supplementary Fig. 2). Collectively these findings suggest vagus nerve stimulation harnesses splenic ChAT-EGFP^+^ T lymphocytes to increase platelet cytosolic calcium via α7nAChR.

### Vagus nerve stimulation enhances α granule release

Platelet activation after an injury is characterized by the fusion of intracellular α granules with the plasma membrane to release prothrombotic proteins, including P-selectin, conformational change to the active primary fibrinogen receptor GPIIb/IIIa that facilitates cross-linking and aggregation, and increased membrane expression of phosphatidylserine that promotes prothrombinase complex formation and clot formation^36–38^. To explore these cellular activation events, we first analyzed circulating platelets from healthy uninjured mice. Compared with sham stimulation, platelets remain quiescent after vagus nerve stimulation without increased expression of P-selectin (Fig. 3c, d, representative image), active GPIIb/IIIa receptor (Supplementary Fig. 3a), or phosphatidylserine (Supplementary Fig. 3b).

We then performed vagus nerve stimulation on uninjured mice and exposed circulating platelets to the calcium-dependent agonists thrombin, adenosine diphosphate (ADP), or collagen^39^. In the presence of thrombin, significantly more platelets express P-selectin after vagus nerve stimulation as compared with sham stimulation (Fig. 3e, f, representative image). In contrast, expression of active GPIIb/IIIa or phosphatidylserine following thrombin exposure remains unchanged after vagus nerve stimulation as compared with sham stimulation (Supplementary Fig. 3c, d). In α7nAChR-deficient mice, vagus nerve stimulation fails to increase P-selectin expression after thrombin exposure as compared with sham stimulation (Fig. 3g). Similarly, in athymic nude (Foxn1^nu^) mice, vagus nerve stimulation fails to increase P-selectin expression after thrombin exposure as compared with sham stimulation (Supplementary Fig. 5a). Compared with sham stimulation, vagus nerve stimulation does not increase expression of active GPIIb/IIIa or phosphatidylserine after thrombin exposure in α7nAChR-deficient (Supplementary Fig. 4a, b) or T cell-deficient (Foxn1^nu^) mice (Supplementary Fig. 5b, c), respectively.

In the presence of ADP, we did not observe any difference in platelet P-selectin expression between vagus nerve stimulation or sham stimulation in wild-type (Supplementary Fig. 3e), α7nAChR-deficient (Supplementary Fig. 4c) or athymic nude (Foxn1^nu^) mice (Supplementary Fig. 5d). Compared with sham stimulation, there is no difference in expression of active GPIIb/IIIa or phosphatidylserine with ADP exposure after vagus nerve stimulation in wild-type (Supplementary Fig. 3f, g), α7nAChR-deficient (Supplementary Fig. 4d, e), or athymic nude (Foxn1^nu^) mice (Supplementary Fig. 5e, f). In the presence of collagen, we did not observe any difference in platelet P-selectin expression between vagus nerve stimulation or sham stimulation in wild-type (Supplementary Fig. 3h), α7nAChR-deficient (Supplementary Fig. 4f), or athymic nude (Foxn1^nu^) mice (Supplementary Fig. 5g). Compared with sham stimulation, there is no difference in expression of active GPIIb/IIIa or phosphatidylserine with collagen exposure after vagus nerve stimulation in wild-type (Supplementary Fig. 3i, j), α7nAChR-deficient (Supplementary Fig. 4g, h), or athymic nude (Foxn1^nu^) mice (Supplementary Fig. 5h, i). Together these findings show that T lymphocytes and α7nAChR are required for vagus nerve stimulation to enhance platelet activation, and vagus nerve stimulation may selectively facilitate thrombin-mediated α granule release.

### Vagus nerve stimulation accelerates local clot formation

Recruitment and subsequent activation of circulating platelets at sites of vascular injury support local thrombin generation that is essential for fibrin deposition, stabilization of developing clots, and bleeding cessation^40,41^. To determine if vagus nerve-primed platelets are sufficient to improve hemostasis, we performed vagus nerve stimulation on uninjured mice and transferred circulating platelets into naïve animals undergoing tail transection. We observed that mice reconstituted with platelets from stimulated mice have significantly shorter bleeding times as compared with mice receiving platelets from sham-stimulated animals (Fig. 3h). Complete blood counts on uninjured donor mice show no significant difference in circulating platelet or other blood cell counts, suggesting that vagus nerve stimulation does not increase absolute platelet number (Table 1). Considering that vagus nerve stimulation accelerates carotid thrombosis and thrombin generation is a hallmark of this injury model^42^, we evaluated thrombin production during tail hemorrhage by measuring thrombin-antithrombin (TAT) complex concentrations. Compared with sham stimulation, vagus nerve stimulation significantly increases local TAT levels at the tail transection site (Fig. 3i). We did not observe a difference in circulating TAT levels between vagus nerve stimulation and sham stimulation (Fig. 3i). Similar to hemophilia A mice, injured wild-type animals demonstrate normal pulmonary architecture without evidence of thromboembolism after vagus nerve stimulation (Fig. 3j, k).

**Table 1:** Complete blood cell counts

	SHAM		VNS		*p* value
	Avg.	SEM	Avg.	SEM	
WBC (K/μL)	2.67	0.70	2.94	1.24	*p* > 0.05
Hb (g/dL)	12.08	0.35	11.58	0.48	*p* > 0.05
Hct (%)	44.03	0.68	42.20	1.19	*p* > 0.05
Plt (K/μL)	758	90.5	724.3	90.9	*p* > 0.05

Data were presented as mean ± s.e.m. (*n* = 4 mice). Statistical significance was determined by unpaired two-tailed Student’s *t*-test. Experiments were performed two times. Source data are provided as a Source Data File.

### Systemic hemodynamics and tissue perfusion remain unchanged

Finally, to determine if the parasympathetic effects of vagus nerve stimulation contribute to hemostasis, we performed electrocardiograms and measured systemic blood pressure in wild-type, α7KO, and Foxn1^nu^ mice. Compared with pre-stimulation, we did not observe any differences in post-stimulation mean heart rate or arterial blood pressure in wild-type (Supplementary Fig. 6a, b), α7KO (Supplementary Fig. 6c, d), or Foxn1^nu^ mice (Supplementary Fig. 6e, f). Furthermore, we did not observe any differences in post-stimulation tail blood flow (Supplementary Data Fig. 6g) or tail blood volume in wild-type mice (Supplementary Fig. 6h). These findings suggest vagus nerve stimulation primes circulating platelets to accelerate local clot formation without inducing systemic thrombosis or influencing systemic hemodynamics or tissue perfusion to the site of injury.

## Discussion

**Fig. 4:**
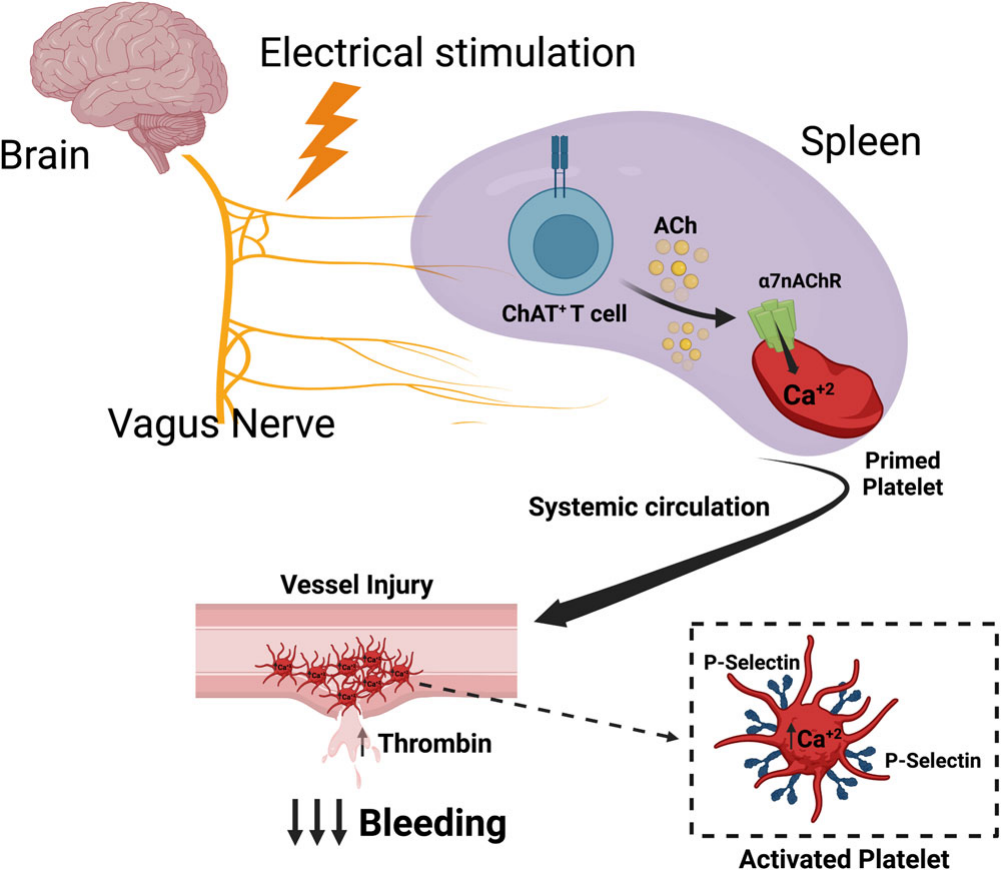
The Neural Tourniquet. Electrical vagus nerve stimulation harnesses acetylcholine-secreting ChAT^+^ T lymphocytes in the spleen to stimulate platelet calcium uptake and alpha granule secretion via an α7nAChR-dependent mechanism. Vagus nerve stimulation primes circulating platelets to increase local thrombin production that accelerates clotting and reduces bleeding at sites of tissue injury.

Taken together, these studies reveal a previously unrecognized neural hemostatic mechanism (Fig. 4). The vagus nerve targets circulating platelets in the spleen that occupy a critical role in systemic hemostasis. Previously implicated in relaying neural signals in the inflammatory reflex, regulating blood pressure and vascular contractility, and controlling cytotoxic T cells and clearance of chronic viral infection, our data implicate acetylcholine-synthesizing ChAT-EGFP^+^ T lymphocytes in the regulation of platelet function and hemostasis following vagus nerve stimulation^27,43–45^. Here we show platelets in direct contact with ChAT-EGFP^+^ T lymphocytes in the spleen. Platelets from T cell-deficient animals fail to respond to vagus nerve stimulation. Vagus nerve stimulation also fails to control bleeding in T cell-deficient mice, but a reconstitution of these animals with ChAT-EGFP^+^ T lymphocytes restores normal hemostasis. Thus ChAT-EGFP^+^ T cells are positioned to enhance platelet function after vagus nerve stimulation. Notwithstanding, the release of other endogenous ligands in the spleen following vagus nerve stimulation may control platelet function. Previous work shows splenic lymphocytes secrete choline acetyltransferase (ChAT), which may synthesize additional extracellular acetylcholine to support platelet α7nAChR signaling^46^. Establishing a critical role for ChAT-EGFP^+^ T lymphocytes in modulating hemorrhage following vagus nerve stimulation provides further insights into how hemostatic and immunologic mechanisms control the host response to injury.

Circulating platelets are quiescent unless exposed to vascular injury, which upregulates coagulation factor activity on their surface to create a stable fibrin clot. Since vagus nerve stimulation increases platelet cytosolic calcium via α7nAChR to facilitate future activation, this priming phenomenon enhances circulating platelet function to extend neural control of hemostasis to sites lacking direct vagal innervation. Our results show that vagus nerve stimulation does not facilitate activation in all platelets, which may relate, in part, to only 60% expressing surface α7nAChR. Because we utilized acute hemorrhage and thrombosis models, it is unclear how long circulating platelets remain in a primed state after vagus nerve stimulation, or if repeated stimulation is required to maintain enhanced platelet function. Ongoing platelet clearance from the circulation may impact hemostasis during chronic vagus nerve stimulation.

Although α7nAChR functions as a traditional ligand-gated ion channel in neuronal cells, it activates G-protein coupled, inositol triphosphate (IP3)-induced calcium release in neuronal and non-neuronal immune cells^47–49^. Our finding that vagus nerve stimulation requires extracellular calcium to increase platelet cytosolic calcium is consistent with cholinergic signaling in human platelets^28^ and suggests canonical α7nAChR ion channels facilitate activation, accelerated thrombosis, and decreased bleeding. Additional studies may determine if metabotropic α7nAChR signaling contributes to platelet activation following vagus nerve stimulation. Vagus nerve stimulation enhances platelet alpha granule release in the presence of thrombin without increasing active GPIIB/IIIA or phosphatidylserine expression. While thrombin, ADP, and collagen induce cytosolic calcium mobilization to promote platelet activation^50–52^, and thrombin can trigger ADP release and activate GPIIb/IIIa^53^, the degree or kinetics of calcium upregulation after vagus nerve stimulation may favor alpha granule release versus other activation pathways^54,55^. For example, phosphatidylserine expression requires sustained, supramaximal concentrations of intracellular calcium^56^. Co-stimulation with collagen and thrombin causes 100% of platelets to express P-selectin, but only 20–40% express phosphatidylserine^57,58^. Increases in basal cytosolic calcium after vagus nerve stimulation may be sufficient to enhance alpha granule release but not phosphatidylserine exposure. Heterogenous platelet activation is observed within hemostatic plugs comprised of core, degranulated platelets expressing P-selectin surrounded by an outer shell of P-selectin-negative cells^59^. Moreover, the growth of the platelet core is dependent on thrombin signaling, whereas interruption of ADP signaling inhibits shell growth independent of the core region^59^. Additional studies may determine if vagus nerve stimulation targets platelets that function within the hemostatic plug core.

The extent of vascular injury governs platelet activation. While superficial lesions that disrupt the subendothelial vessel layer invoke ADP receptor pathways, GPIIb/IIIa, and GPVI-collagen, injury to deeper vessel layers elicits stronger clotting responses involving thrombin-driven pathways^60^. Interestingly, von Willebrand factor (vWF)-deficient mice demonstrate impaired thrombosis after both types of injuries^60^. The binding of subendothelial vWF to platelet GPIbα at the site of intimal injury supports platelet adhesion and may increase subsequent platelet aggregation^61^. GPIbα is the most heavily glycosylated platelet surface protein, with sialic acid-capped carbohydrate chains contributing to 64% of the total sialic acid content^61^. Li et al. found that monoclonal antibodies to GPIbα increase P-selectin expression, activation of GPIIb/IIIa, and glycoprotein desialylation^61^. Considering that vagus nerve stimulation increases platelet P-selectin expression without activating GPIIb/IIIa, GPIbα-mediated pathways may not be required. Consistent with our findings, Sambrano et al. demonstrated that protease-activated receptor (PAR) 4 knockout mice with deficient thrombin signaling bleed significantly longer after tail transection and have prolonged clot formation after ferric chloride-induced vessel injury, indicating that platelet activation by thrombin is essential for normal hemostasis^62^. Thus vagus nerve stimulation may facilitate thrombin-mediated platelet activation to respond to more severe injuries. Further studies may elucidate the contribution of PAR receptors to hemostasis following vagus nerve stimulation.

Vagus nerve stimulation is being studied for the treatment of medically-refractory depression, epilepsy, rheumatoid arthritis, and Crohn’s disease^63–66^. Since 1997, more than 200,000 patients have received vagus nerve stimulation via implantable pulse generators, an FDA-approved therapy that is safe and well tolerated^67^. Noninvasive techniques to stimulate the vagus nerve are also under development, including methods using focused ultrasound^68,69^. This approach may allow for emergent treatment of traumatic hemorrhage in individuals not fitted with implantable devices. Noninvasive vagus nerve stimulation of blood donors may improve platelet function in transfusion recipients. Vagus nerve stimulation may reduce overall transfusion volumes, minimize transfusion-related complications and costs, and ease the burden on blood bank stores^70,71^. Because hemophilia A affects hundreds of thousands of individuals and causes significant morbidity and mortality, it will be interesting to further study the role of vagus nerve signaling for this and other bleeding disorders^72^.

## Methods

### Mice

Adult male 8–12-week-old BALB/c mice (20–25 g, Taconic), adult male 8–12-week-old C57BL6/J mice (20–25 g, Jackson Labs), adult male 8–16-week-old α7nAChR-deficient mice (20–25 g, Jackson Labs, C57BL6/J background) and wild-type littermates, adult male 8–12-week-old (Foxn1^nu^) nude mice (20–25 g Taconic, BALB/c background), adult male 8–12-week-old factor VIII-knockout mice (20–25 g, Jackson Labs), adult male 8–12-week-old ChAT(BAC)-EGFP mice (20–25 g, Jackson Labs), and adult male 8–12-week-old ChAT-TdTomato mice generated from crossing ChAT-Cre mice (B6;129S6-Chattm2(cre)Lowl/J, Jackson Labs #006410) with Td-Tomato mice (B6.Cg-Gt(ROSA)26Sortm14(CAG-tdTomato)Hze/J, Jackson Labs #007914) expressing red fluorescent protein in cholinergic cells are used for experiments. All animals are housed at 22 °C (range 20–26 °C) and 42% humidity (range 30–70%) on a 12-h light/dark cycle. Foxn1^nu^ nude mice are housed in a room devoted to immunocompromised animals. Standard animal chow and water are freely available. Food and water are not withheld before experiments. Following survival surgery, animals are provided a warming blanket and administered 0.9% normal saline (30–50 mL/kg, s.c.). Animals return to normal housing after achieving sternal recumbency and ambulation. Animals are monitored twice daily for 72 h post-operatively and given analgesia (Buprenex 0.1 mL/kg, s.c.) at each visit. After 72 h, animals are monitored twice weekly until the termination of the experiment. Humane endpoints for early euthanasia include the inability to stand, agonal, rapid, or labored breathing, significant decreases in activity score, sick posturing, decreased grooming, or reduced response to touch. Animals are euthanized by gradual displacement CO2 asphyxiation and terminal exsanguination under anesthesia. Endpoints for early anesthesia for bleeding experiments include lethal blood loss, increased pallor, drying of mucous membranes, or writhing. All animal experiments are performed in accordance with the National Institutes of Health (NIH) Guidelines under protocols approved by the Institutional Animal Care and Use Committee of The Feinstein Institutes for Medical Research.

### Vagus nerve stimulation

Animals are anesthetized with ketamine (144 mg/kg, i.p.) and xylazine (14 mg/kg, i.p.). After seven minutes, animals are placed in the supine position, and a 1–2 cm ventral midline cervical skin incision is made between the mandible and sternum. The subcutaneous tissue and mandibular salivary glands are dissected and retracted laterally. The left vagus nerve is isolated from between the sternomastoid and sternohyoid muscles, dissected free from the neighboring carotid artery, and controlled with a 5-0 prolene suture. The nerve is then mounted on bipolar platinum electrodes (Plastics One). Constant voltage stimuli (1 V, 30 Hz, 2 ms pulse width) are applied for five minutes. Electrical stimuli are generated using an MP36R Data Acquisition System (Biopac Systems, Goleta, CA) attached to an out three low voltage stimulation adapter. Sham-stimulated animals receive cervical skin incision and dissection of the salivary glands, but the vagus nerve is neither isolated nor dissected free from neighboring structures.

### Tail transection and hemorrhage

Mice are screened before study inclusion to ensure visual similarity and consistency between the anatomy of the tail tips and the site of transection. Any animals displaying aberrant anatomy and/or injury that does not meet our predetermined criteria are excluded. Following vagus nerve stimulation or sham surgery, tails are immersed in water at 37 ± 1 °C for 5 min. Tails are then removed from the solution, amputated 2 mm from the tip with a razor blade, and immediately placed into a 50 mL beaker containing water at 37 ± 1 °C. Tails are allowed to bleed uncontrolled until the bleeding stops for a minimum of ten seconds. This duration of bleeding is recorded as bleeding time. For analyses of bleeding in factor VIII deficient mice, tails are first immersed in 0.9% saline at 37 ± 1 °C for 5 min. Tails are then removed and amputated 2 mm from the tip with a razor blade. Tails are then placed into a 50 mL conical tube containing 0.9% saline at 37 ± 1 °C. Tails are allowed to bleed freely for a total of 10 min. Total blood loss is determined by densitometry by measuring absorbance at 550 nm. A standard curve is created from a known volume of blood.

### Ferric chloride-induced carotid artery injury

Animals are anesthetized with isoflurane (1–2%), placed in the supine position, and a 1–2 cm ventral midline cervical skin incision is made between the mandible and sternum. The left vagus nerve is isolated from the carotid sheath and placed on a bipolar electrode and stimulated (1 V, 30 Hz, 2 ms pulse width, 5 min). Following stimulation or sham surgery, the right carotid artery is dissected free from surrounding tissue for a distance of 5 mm, and the artery is bathed in 5 μL of a 10% ferric chloride (FeCl_3_) solution for 3 min (F8KO mice) or a 5% FeCl_3_ solution for 1 min (C57BL6/J and α7KO mice). The carotid artery is then rinsed with 0.9% normal saline. A doppler ultrasound probe (L8–18i-RS, GE) attached to a GE Logiq e ultrasound system is placed over both carotid arteries to monitor blood flow for a total of 25 min. Experimental endpoints include cessation of blood flow for >1 min as determined by the absence of arterial waveform on motion mode (m-mode) and doppler signal as compared to the contralateral, unaffected carotid artery. If the occlusion is not observed after 25 min, the time is recorded as 25 min for statistical comparisons.

### Splenectomy

Animals are anesthetized with ketamine (144 mg/kg, i.p.) and xylazine (14 mg/kg, i.p.). Animals are placed in the supine position, and a 2 cm laparotomy incision is made with a scalpel. The spleen is exteriorized and the splenic artery and vein are ligated with a 5-0 prolene suture. The peritoneum is closed with 5-0 Vicryl simple interrupted sutures and the skin with a 7 mm skin stapler. Sham-splenectomized animals undergo midline laparotomy only. Splenectomy or sham-splenectomy is performed 6 weeks before tail transection.

### Heart rate, blood pressure, tail blood flow, and volume recording

Animals are anesthetized with ketamine (144 mg/kg, i.p.) and xylazine (14 mg/kg, i.p.) and placed on a heating pad set at 37 °C and left to thermoregulate for 7 min. Blood pressure, heart rate, tail blood flow, and tail blood volume are measured simultaneously and non-invasively using the tail-cuff method with the CODA Monitor NonInvasive Blood Pressure System (Kent Scientific)^73^. The occlusion cuff is positioned near the base of the tail, followed by the volume pressure recording (VPR) cuff positioned adjacent and distal to the occlusion cuff. To assure the ideal temperature before recordings, the tail temperature is recorded with an infrared thermometer at the base, with the entire tail lying on the heating pad. Recordings are conducted with a fully automated occlusion tail cuff and consist of 15 cycles (five of which are acclimatization cycles) with a deflation time of 20 and 10 s between each cycle. Measurements from ten consecutive cycles are recorded as a baseline. Following baseline recordings, a 1–2 cm ventral midline cervical skin incision is made and the left vagus nerve is isolated and controlled as described previously. The nerve is mounted on bipolar platinum electrodes (Plastics One) and constant voltage stimulation (1 V, 30 Hz, 2 ms pulse width) is applied for 5 min. Following vagus nerve stimulation, animals are allowed to thermoregulate on a heating pad set at 37 °C for an additional 5 min and recordings post-stimulation are performed for another ten consecutive cycles.

### Platelet receptor analysis

Systemic blood from the inferior vena cava is collected 17 min after vagus nerve stimulation (1 V, 30 Hz, 2 ms, 5 min) into Tris-buffered saline (20 mM Tris-HCl, 137 mM NaCl, pH 7.3) containing 20 U/mL heparin. Heparinized whole blood is then diluted with modified Tyrode’s Buffer (134 mM NaCl, 0.34 mM Na_2_HPO_4_, 2.9 mM KCl, 12 mM NaHCO_3_ 20 mM Hepes, pH 7.0 with 5 mM glucose, 0.35% BSA). On flow cytometry (BD FACSymphony A3, BD LSRFortessa), platelets are initially identified via forward and side scatter plots (FSC-A/SSC-A) followed by anti-CD41a rat monoclonal antibody (Phycoerythrin/Cy7 clone: MWReg30, Cat No. 133916, BioLegend) (Supplementary Fig. 7a, b). Platelets are then stimulated ex vivo with thrombin (1 U/ml, Sigma-Aldrich), collagen (0.5 or 5 μg/ml, Sigma-Aldrich), or ADP (20 μg/ml, Thermo Scientific), and stained with mouse monoclonal antibody against CD62P (P-Selectin) (APC Allophycocyanin clone: RMP-1, Cat No. 148304, BioLegend), active GPIIb/IIIa (CD41/CD61-PE, Cat No. M023-2, Emfret Analytics), and phosphatidylserine (Clone 1H6, Alexa Fluor 488, Cat No. 16–256, Millipore Sigma). All antibodies are applied at 1:20 (final dilution). In separate experiments, non-stimulated platelets are stained with anti-nicotinic acetylcholine receptor α7 (CHRNA7) (extracellular)-FITC antibody (Alomone Labs). Flow cytometry data is then collected (BD FACSDiva685 Software) and analyzed (FlowJo 10.8.1).

### Thrombin generation

Animals receive vagus nerve stimulation followed by tail warming in a 37 °C water bath for 5 min. The tail is transected where its diameter was 3 mm, and allowed to bleed freely into citrated tubes to collect local shed blood. Systemic blood from the inferior vena cava is then collected directly into citrated tubes. Blood is centrifuged for 15 min at 1500×*g* and plasma stored at −80 °C until assay. Thrombin generation is determined by measurement of the thrombin-antithrombin (TAT) complex via commercially available ELISA (Abcam, Cambridge, MA).

### Factor VIII activity

Animals receive vagus nerve stimulation (5 min) or sham stimulation followed by tail warming in a 37 °C water bath for 5 min. Systemic blood is collected via cardiac puncture after 7 min. Blood is anticoagulated with sodium citrate and centrifuged at 1500×*g* for 15 min. Platelet-poor plasma is stored at −80 °C until assay. Factor VIII activity is determined via standard clinical assay.

### Lung histology

Following tail transection and bleeding time measurement, lung tissue is collected. Briefly, lungs are inflated with 10% formalin by atmospheric pressure, excised, embedded in paraffin, sectioned at 6–7 µm and stained with hematoxylin and eosin. A board-certified thoracic pathologist blinded to the treatment assesses histologic sections for the presence of microthrombi and inflammation. Pictomicrographs are collected with a Zeiss Apotome connected to a computer with Zeiss AxioVision installed (Carl Zeiss Microscopy GmbH, Jena, Germany).

### Platelet adoptive transfer

Male aged 8–12-week-old C57BL/6 mice (Taconic Biosciences, Hudson, NY) are anesthetized with ketamine/xylazine and receive vagus nerve stimulation (1 V, 30 Hz, 2 ms, 5 min) or sham stimulation as described above. After 17 min, systemic blood is collected from the inferior vena cava into Tris buffer saline containing 20 U/mL Heparin. Heparinized blood is centrifuged for 5 min at 500 × *g*. Platelet-rich plasma (PRP) is transferred to a clean tube and centrifuged again for 8 min at 300 × *g*. PRP of all mice from each stimulation group is combined and centrifuged for 5 min at 1300 × *g*. The platelet pellet is suspended in 0.9% normal saline with 5% BSA. The final platelet concentration is determined by flow cytometry. Male aged 8–12-week-old C57BL/6 mice are anesthetized with ketamine/xylazine, receive donor platelets (6 × 10^8^ platelets/200 µL, r.o.), and after 9 min undergo tail transection.

### ChAT-eGFP^+^ T-cell adoptive transfer

Transgenic ChAT^BAC^-eGFP mice (The Jackson Laboratory, Bar Harbor, ME) are euthanized via CO_2_ asphyxiation followed by splenic harvest. Spleens are passed through a 40 µm cell strainer, followed by red blood cell lysis with ACK buffer (Lonza, Allendale, NJ), and then passage through a CD4^+^ negative selection column (Miltenyi Biotech, Bergisch Gladbach, Germany). Eluted cells are treated with Fc block, anti-CD19 (BD Pharmingen, San Jose, CA) and anti-CD62L (eBioscience, San Diego, CA). Stained cells are then negatively selected for these markers by FACS, where this sub-population is divided into ChAT-eGFP^+^ and ChAT-eGFP^-^. Each population is suspended in 0.9% normal saline with 5% BSA and 150,000 cells are injected into the peritoneum of males aged 8–12-week-old (Foxn1^nu^) nude mice (Taconic Biosciences, Hudson, NY). After 5 days, recipient (Foxn1^nu^) nude mice receive vagus nerve stimulation (1 V, 30 Hz, 2 ms, 5 min) or sham stimulation before tail transection.

### Platelet Ca^2+^ flux

Male aged 8–12-week-old C57BL/6 mice (Taconic Biosciences, Hudson, NY) are anesthetized with ketamine/xylazine and receive vagus nerve stimulation (1 V, 30 Hz, 2 ms, 5 min) or sham stimulation as described above. Harvested circulating platelets from the inferior vena cava are washed and loaded with Oregon Green^TM^ BAPTA-1, AM and Fura Red^TM^, AM. Platelets are resuspended in Tyrode’s buffer containing 2 mM Ca^2+^ and baseline calcium concentrations are measured by flow cytometry.

### Immunohistochemistry

Spleens from ChAT-TdTomato expressing mice are excised and fixed in 4% PFA solution at 4 °C overnight and then transferred to 30% sucrose solution. Following embedding in OCT, tissue sections (10 μm) are prepared on a Leica CM1850 cryostat. Splenocytes are incubated in a blocking solution consisting of 10% Normal donkey serum (Southern Biotech, 0030-01) or 10% Normal goat serum (Vector Laboratories, S-1000-20), 0.01% Mouse BD Fc block (BD Bioscience, 553142), and 0.3% Triton X-100 in 1X PBS for 1 h at room temperature. Next, tissues are incubated with primary antibodies: Mouse CD4 Monoclonal Antibody (Thermo Scientific, MA17631, 1:100) and Rat Anti-CD41 antibody (Abcam, ab33661, 1:250) diluted in 0.3% Triton-100, 0.01% Mouse BD Fc block, and 10% NDS or NGS with 1X PBS overnight at 4 °C. Tissues are rinsed with 10x PBS-Tween 20 (0.1 M PBS, 0.5% Tween 20, pH 7.4) and incubated with secondary antibodies: Donkey anti-mouse Dylight 488 (Abcam, ab96675, 1:250) and Donkey anti-rat Alexa Fluor 647 (Invitrogen, #A48272, 1:250) diluted in 0.3% Triton-100, 0.01% Mouse BD Fc block, and 10% NDS or NGS with 1X PBS overnight at 4 °C. Lastly, tissues are rinsed with 10x PBS-Tween 20 and coverslipped with DAPI Fluoromount Mounting Medium (Southern Biotech, Birmingham, AL, US). Fluorescent images are captured with a Zeiss LSM 900 confocal microscope. Zeiss ZEN black and blue software are used to collect data and process images.

### Cell counts

Animals are anesthetized with ketamine (144 mg/kg, i.p.) and xylazine (14 mg/kg, i.p.) as described above. Circulating blood from the inferior vena cava is collected seven minutes after vagus nerve stimulation or sham stimulation, placed into standard EDTA tubes, and complete blood count is determined using a Cell-Dyn 3700 Blood Analyzer.

### Statistical analysis and reproducibility

All data were presented as mean ± s.e.m, and *p* < 0.05 is considered significant. *n* represents the number of animals in each experiment, as detailed in the figure legends. Animals are randomly allocated into experimental groups. All measurements are taken from distinct samples, where appropriate. Statistical significance is determined by one-way ANOVA for three groups or unpaired, two-tailed Student’s *t*-test for two groups. The Tukey method is used to adjust for multiple comparisons. Statistical analyses are performed and data were presented using GraphPad Prism 8 (GraphPad Software), BD FACSDiva software (BD Biosciences), and FlowJo 10 (LLC). Each experiment is performed independently two times unless otherwise indicated.

### Reporting summary

Further information on research design is available in the Nature Portfolio Reporting Summary linked to this article.

## Supplementary information

Supplementary Information

Reporting Summary

## Data Availability

All data supporting the findings described in this manuscript are available in the article and its Supplementary Information files. There are no third-party data. Source data are provided with this paper.

## Source data

Source data File
