# Supplementary material for: Vagus nerve stimulation primes platelets and reduces bleeding in hemophilia A male mice
Source: Nat Commun. 2023 Jun 1;14:3122. doi: 10.1038/s41467-023-38505-6 (PMC10235098; doi:10.1038/s41467-023-38505-6)
Supplement: Supplementary file 1 — Supplementary Information [file 41467_2023_38505_MOESM1_ESM.pptx]

## Slide 1
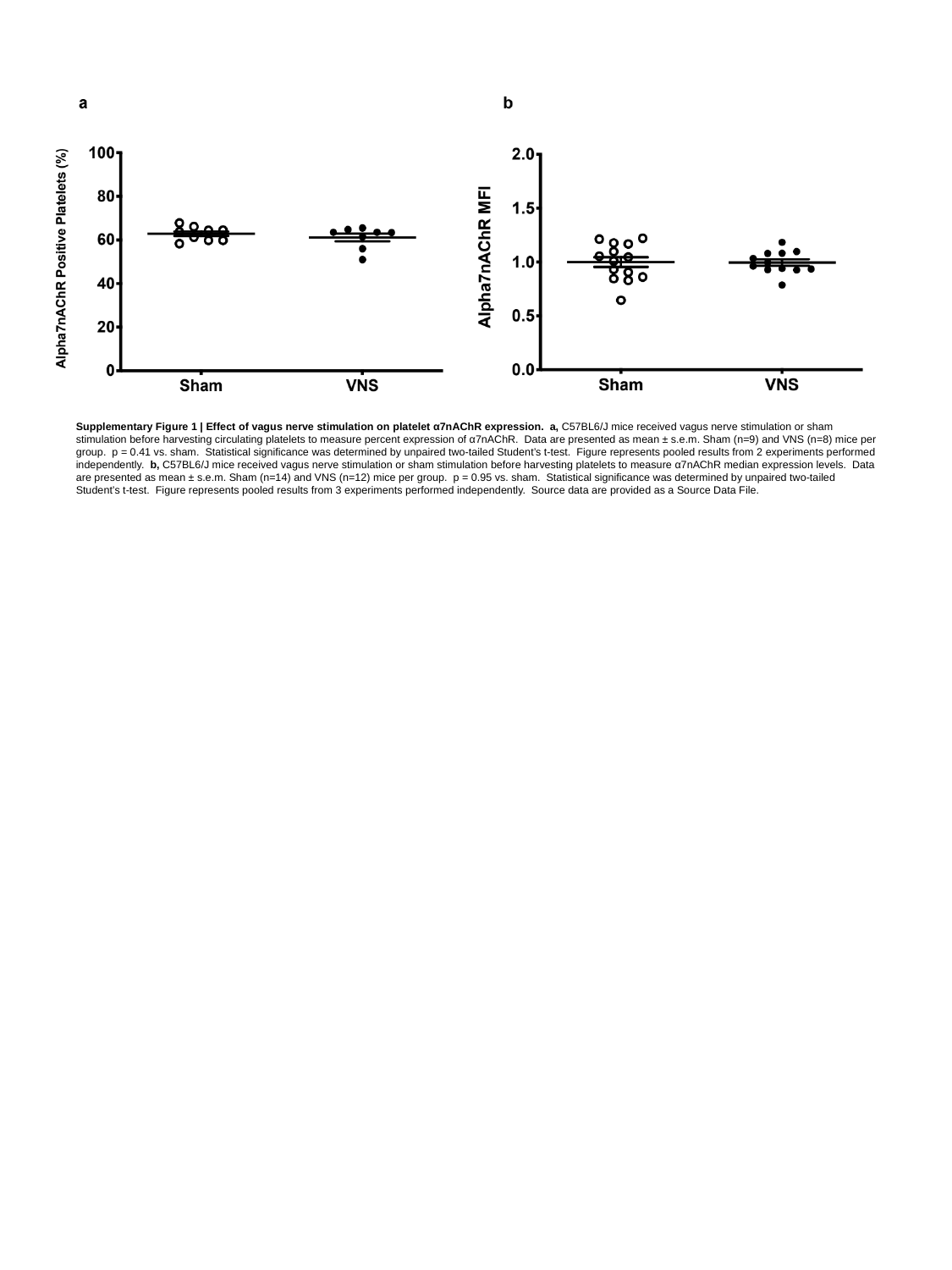

Supplementary Figure 1 | Effect of vagus nerve stimulation on platelet α7nAChR expression. a, C57BL6/J mice received vagus nerve stimulation or sham stimulation before harvesting circulating platelets to measure percent expression of α7nAChR. Data are presented as mean ± s.e.m. Sham (n=9) and VNS (n=8) mice per group. p = 0.41 vs. sham. Statistical significance was determined by unpaired two-tailed Student’s t-test. Figure represents pooled results from 2 experiments performed independently. b, C57BL6/J mice received vagus nerve stimulation or sham stimulation before harvesting platelets to measure α7nAChR median expression levels. Data are presented as mean ± s.e.m. Sham (n=14) and VNS (n=12) mice per group. p = 0.95 vs. sham. Statistical significance was determined by unpaired two-tailed Student’s t-test. Figure represents pooled results from 3 experiments performed independently. Source data are provided as a Source Data File.

## Slide 2
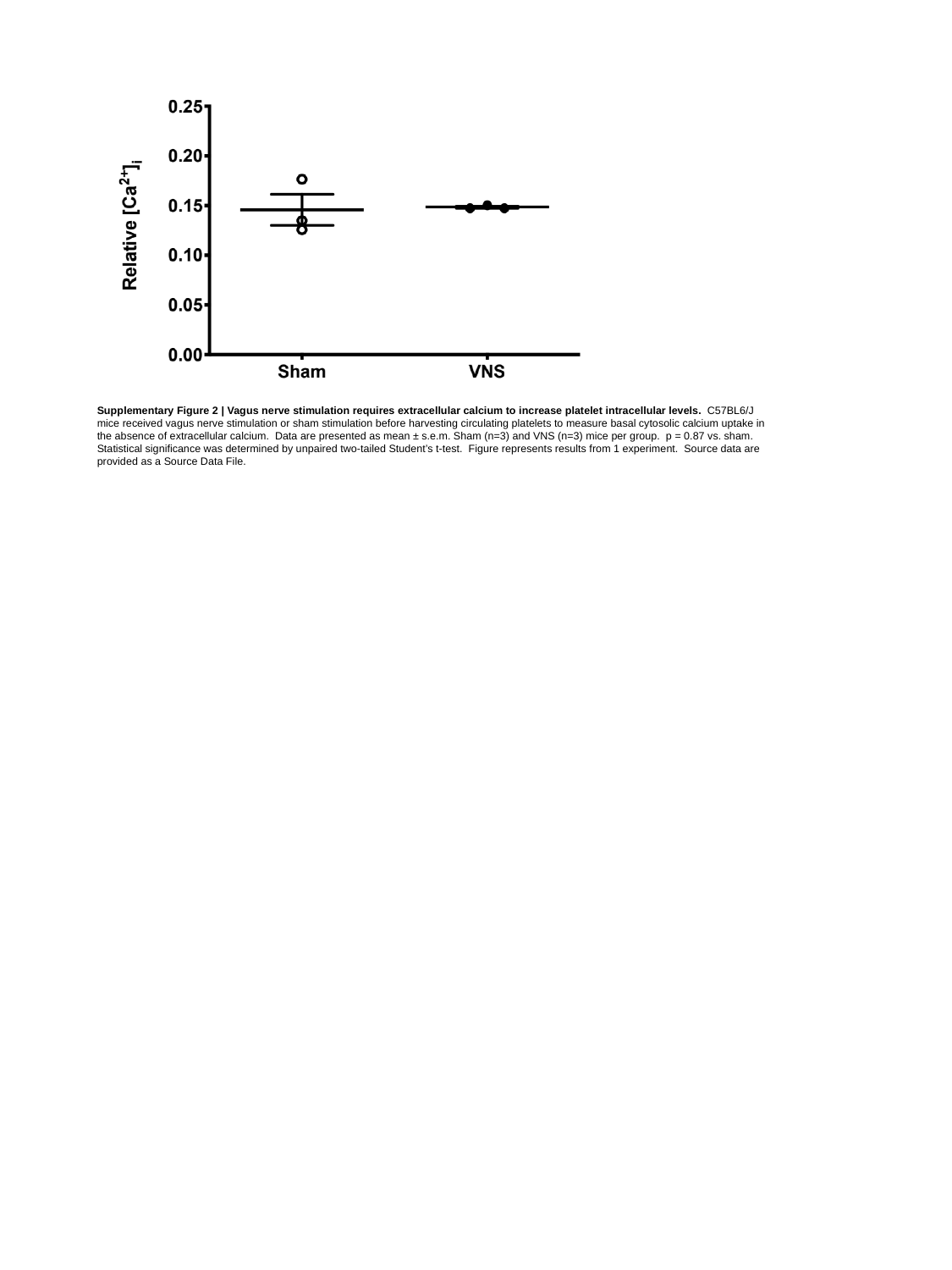

Supplementary Figure 2 | Vagus nerve stimulation requires extracellular calcium to increase platelet intracellular levels. C57BL6/J mice received vagus nerve stimulation or sham stimulation before harvesting circulating platelets to measure basal cytosolic calcium uptake in the absence of extracellular calcium. Data are presented as mean ± s.e.m. Sham (n=3) and VNS (n=3) mice per group. p = 0.87 vs. sham. Statistical significance was determined by unpaired two-tailed Student’s t-test. Figure represents results from 1 experiment. Source data are provided as a Source Data File.

## Slide 3
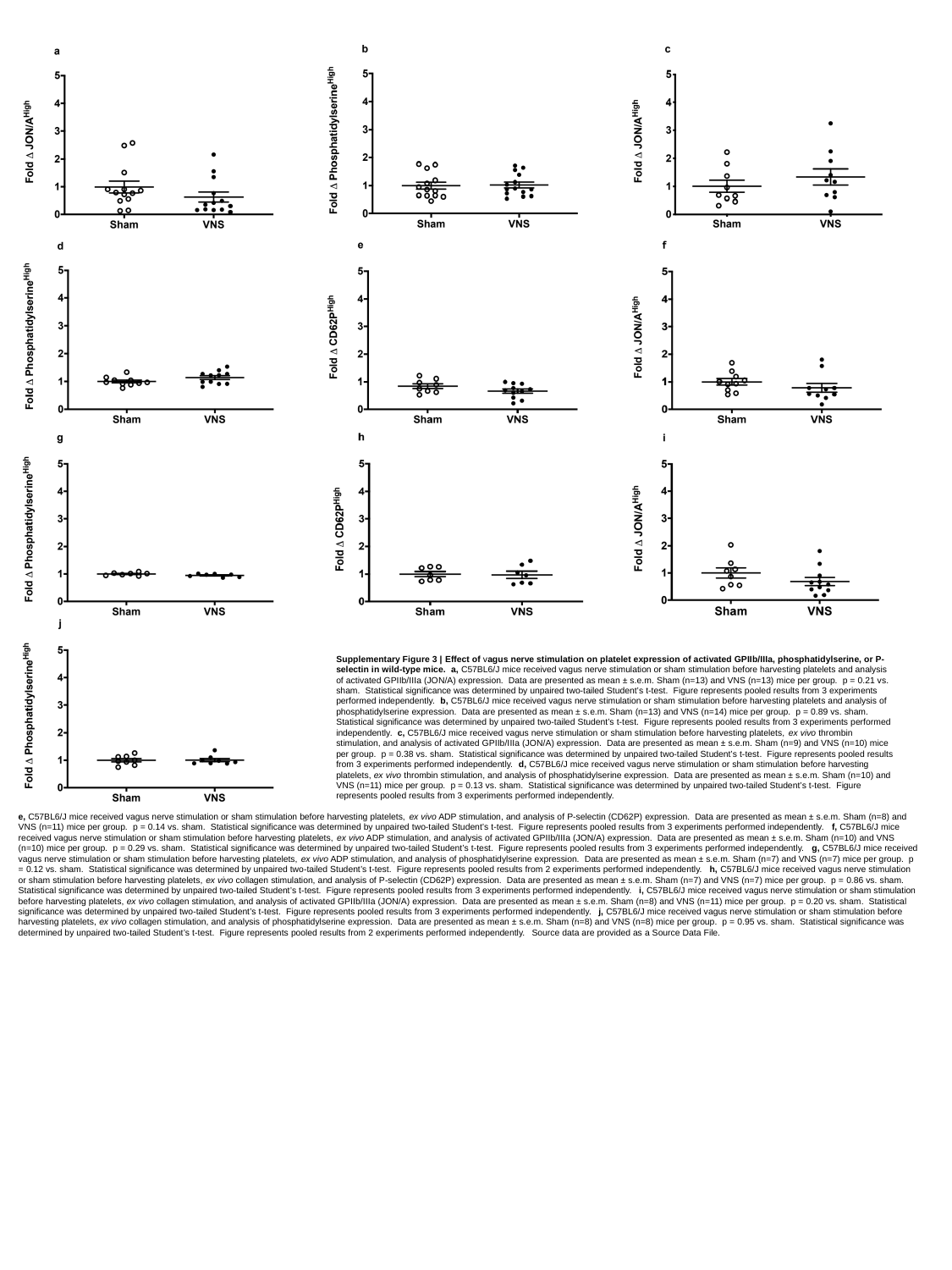

Supplementary Figure 3 | Effect of vagus nerve stimulation on platelet expression of activated GPIIb/IIIa, phosphatidylserine, or P-selectin in wild-type mice. a, C57BL6/J mice received vagus nerve stimulation or sham stimulation before harvesting platelets and analysis of activated GPIIb/IIIa (JON/A) expression. Data are presented as mean ± s.e.m. Sham (n=13) and VNS (n=13) mice per group. p = 0.21 vs. sham. Statistical significance was determined by unpaired two-tailed Student’s t-test. Figure represents pooled results from 3 experiments performed independently. b, C57BL6/J mice received vagus nerve stimulation or sham stimulation before harvesting platelets and analysis of phosphatidylserine expression. Data are presented as mean ± s.e.m. Sham (n=13) and VNS (n=14) mice per group. p = 0.89 vs. sham. Statistical significance was determined by unpaired two-tailed Student’s t-test. Figure represents pooled results from 3 experiments performed independently. c, C57BL6/J mice received vagus nerve stimulation or sham stimulation before harvesting platelets, ex vivo thrombin stimulation, and analysis of activated GPIIb/IIIa (JON/A) expression. Data are presented as mean ± s.e.m. Sham (n=9) and VNS (n=10) mice per group. p = 0.38 vs. sham. Statistical significance was determined by unpaired two-tailed Student’s t-test. Figure represents pooled results from 3 experiments performed independently. d, C57BL6/J mice received vagus nerve stimulation or sham stimulation before harvesting platelets, ex vivo thrombin stimulation, and analysis of phosphatidylserine expression. Data are presented as mean ± s.e.m. Sham (n=10) and VNS (n=11) mice per group. p = 0.13 vs. sham. Statistical significance was determined by unpaired two-tailed Student’s t-test. Figure represents pooled results from 3 experiments performed independently.
e, C57BL6/J mice received vagus nerve stimulation or sham stimulation before harvesting platelets, ex vivo ADP stimulation, and analysis of P-selectin (CD62P) expression. Data are presented as mean ± s.e.m. Sham (n=8) and VNS (n=11) mice per group. p = 0.14 vs. sham. Statistical significance was determined by unpaired two-tailed Student’s t-test. Figure represents pooled results from 3 experiments performed independently. f, C57BL6/J mice received vagus nerve stimulation or sham stimulation before harvesting platelets, ex vivo ADP stimulation, and analysis of activated GPIIb/IIIa (JON/A) expression. Data are presented as mean ± s.e.m. Sham (n=10) and VNS (n=10) mice per group. p = 0.29 vs. sham. Statistical significance was determined by unpaired two-tailed Student’s t-test. Figure represents pooled results from 3 experiments performed independently. g, C57BL6/J mice received vagus nerve stimulation or sham stimulation before harvesting platelets, ex vivo ADP stimulation, and analysis of phosphatidylserine expression. Data are presented as mean ± s.e.m. Sham (n=7) and VNS (n=7) mice per group. p = 0.12 vs. sham. Statistical significance was determined by unpaired two-tailed Student’s t-test. Figure represents pooled results from 2 experiments performed independently. h, C57BL6/J mice received vagus nerve stimulation or sham stimulation before harvesting platelets, ex vivo collagen stimulation, and analysis of P-selectin (CD62P) expression. Data are presented as mean ± s.e.m. Sham (n=7) and VNS (n=7) mice per group. p = 0.86 vs. sham. Statistical significance was determined by unpaired two-tailed Student’s t-test. Figure represents pooled results from 3 experiments performed independently. i, C57BL6/J mice received vagus nerve stimulation or sham stimulation before harvesting platelets, ex vivo collagen stimulation, and analysis of activated GPIIb/IIIa (JON/A) expression. Data are presented as mean ± s.e.m. Sham (n=8) and VNS (n=11) mice per group. p = 0.20 vs. sham. Statistical significance was determined by unpaired two-tailed Student’s t-test. Figure represents pooled results from 3 experiments performed independently. j, C57BL6/J mice received vagus nerve stimulation or sham stimulation before harvesting platelets, ex vivo collagen stimulation, and analysis of phosphatidylserine expression. Data are presented as mean ± s.e.m. Sham (n=8) and VNS (n=8) mice per group. p = 0.95 vs. sham. Statistical significance was determined by unpaired two-tailed Student’s t-test. Figure represents pooled results from 2 experiments performed independently. Source data are provided as a Source Data File.

## Slide 4
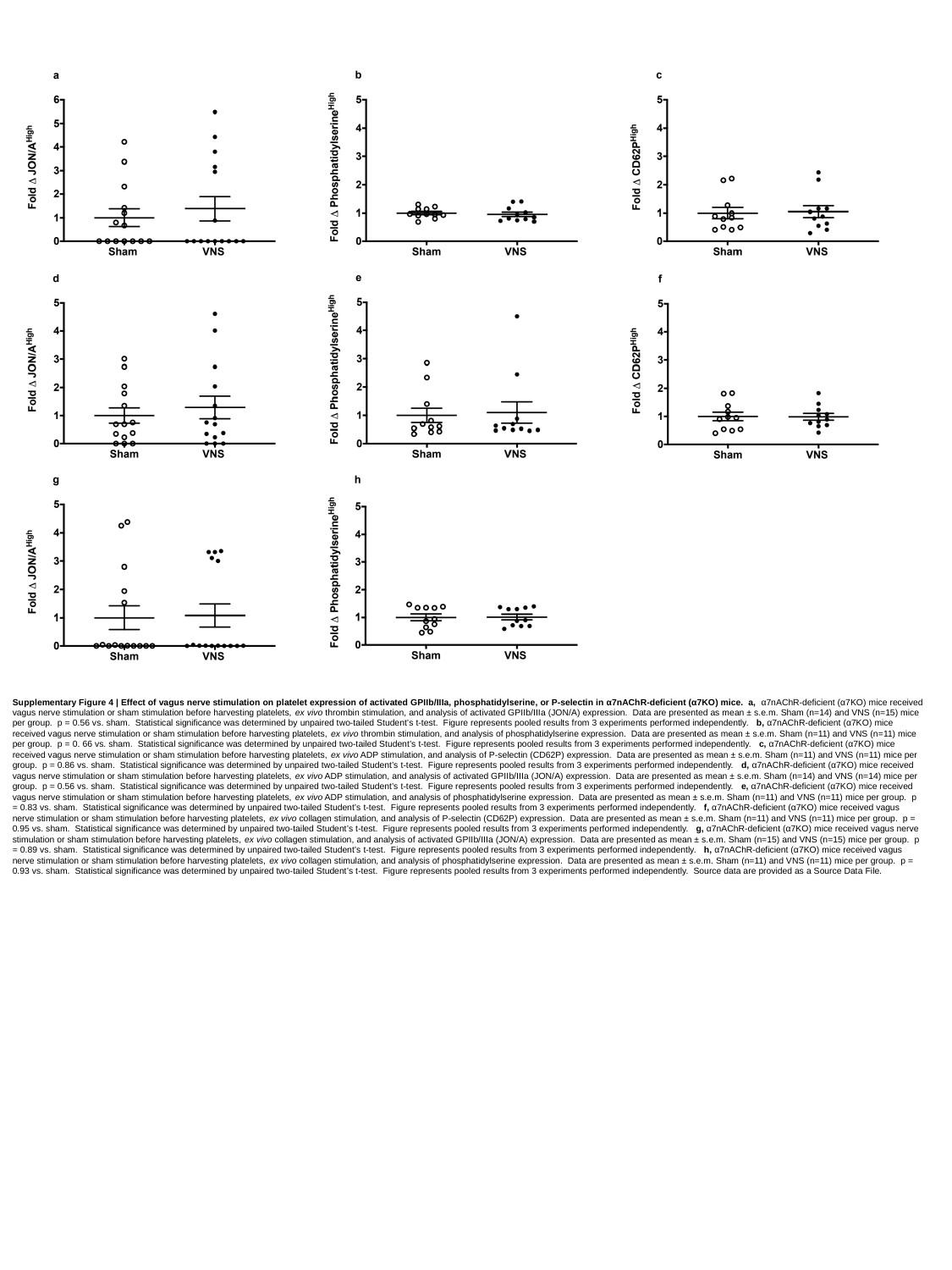

Supplementary Figure 4 | Effect of vagus nerve stimulation on platelet expression of activated GPIIb/IIIa, phosphatidylserine, or P-selectin in α7nAChR-deficient (α7KO) mice. a, α7nAChR-deficient (α7KO) mice received vagus nerve stimulation or sham stimulation before harvesting platelets, ex vivo thrombin stimulation, and analysis of activated GPIIb/IIIa (JON/A) expression. Data are presented as mean ± s.e.m. Sham (n=14) and VNS (n=15) mice per group. p = 0.56 vs. sham. Statistical significance was determined by unpaired two-tailed Student’s t-test. Figure represents pooled results from 3 experiments performed independently. b, α7nAChR-deficient (α7KO) mice received vagus nerve stimulation or sham stimulation before harvesting platelets, ex vivo thrombin stimulation, and analysis of phosphatidylserine expression. Data are presented as mean ± s.e.m. Sham (n=11) and VNS (n=11) mice per group. p = 0. 66 vs. sham. Statistical significance was determined by unpaired two-tailed Student’s t-test. Figure represents pooled results from 3 experiments performed independently. c, α7nAChR-deficient (α7KO) mice received vagus nerve stimulation or sham stimulation before harvesting platelets, ex vivo ADP stimulation, and analysis of P-selectin (CD62P) expression. Data are presented as mean ± s.e.m. Sham (n=11) and VNS (n=11) mice per group. p = 0.86 vs. sham. Statistical significance was determined by unpaired two-tailed Student’s t-test. Figure represents pooled results from 3 experiments performed independently. d, α7nAChR-deficient (α7KO) mice received vagus nerve stimulation or sham stimulation before harvesting platelets, ex vivo ADP stimulation, and analysis of activated GPIIb/IIIa (JON/A) expression. Data are presented as mean ± s.e.m. Sham (n=14) and VNS (n=14) mice per group. p = 0.56 vs. sham. Statistical significance was determined by unpaired two-tailed Student’s t-test. Figure represents pooled results from 3 experiments performed independently. e, α7nAChR-deficient (α7KO) mice received vagus nerve stimulation or sham stimulation before harvesting platelets, ex vivo ADP stimulation, and analysis of phosphatidylserine expression. Data are presented as mean ± s.e.m. Sham (n=11) and VNS (n=11) mice per group. p = 0.83 vs. sham. Statistical significance was determined by unpaired two-tailed Student’s t-test. Figure represents pooled results from 3 experiments performed independently. f, α7nAChR-deficient (α7KO) mice received vagus nerve stimulation or sham stimulation before harvesting platelets, ex vivo collagen stimulation, and analysis of P-selectin (CD62P) expression. Data are presented as mean ± s.e.m. Sham (n=11) and VNS (n=11) mice per group. p = 0.95 vs. sham. Statistical significance was determined by unpaired two-tailed Student’s t-test. Figure represents pooled results from 3 experiments performed independently. g, α7nAChR-deficient (α7KO) mice received vagus nerve stimulation or sham stimulation before harvesting platelets, ex vivo collagen stimulation, and analysis of activated GPIIb/IIIa (JON/A) expression. Data are presented as mean ± s.e.m. Sham (n=15) and VNS (n=15) mice per group. p = 0.89 vs. sham. Statistical significance was determined by unpaired two-tailed Student’s t-test. Figure represents pooled results from 3 experiments performed independently. h, α7nAChR-deficient (α7KO) mice received vagus nerve stimulation or sham stimulation before harvesting platelets, ex vivo collagen stimulation, and analysis of phosphatidylserine expression. Data are presented as mean ± s.e.m. Sham (n=11) and VNS (n=11) mice per group. p = 0.93 vs. sham. Statistical significance was determined by unpaired two-tailed Student’s t-test. Figure represents pooled results from 3 experiments performed independently. Source data are provided as a Source Data File.

## Slide 5
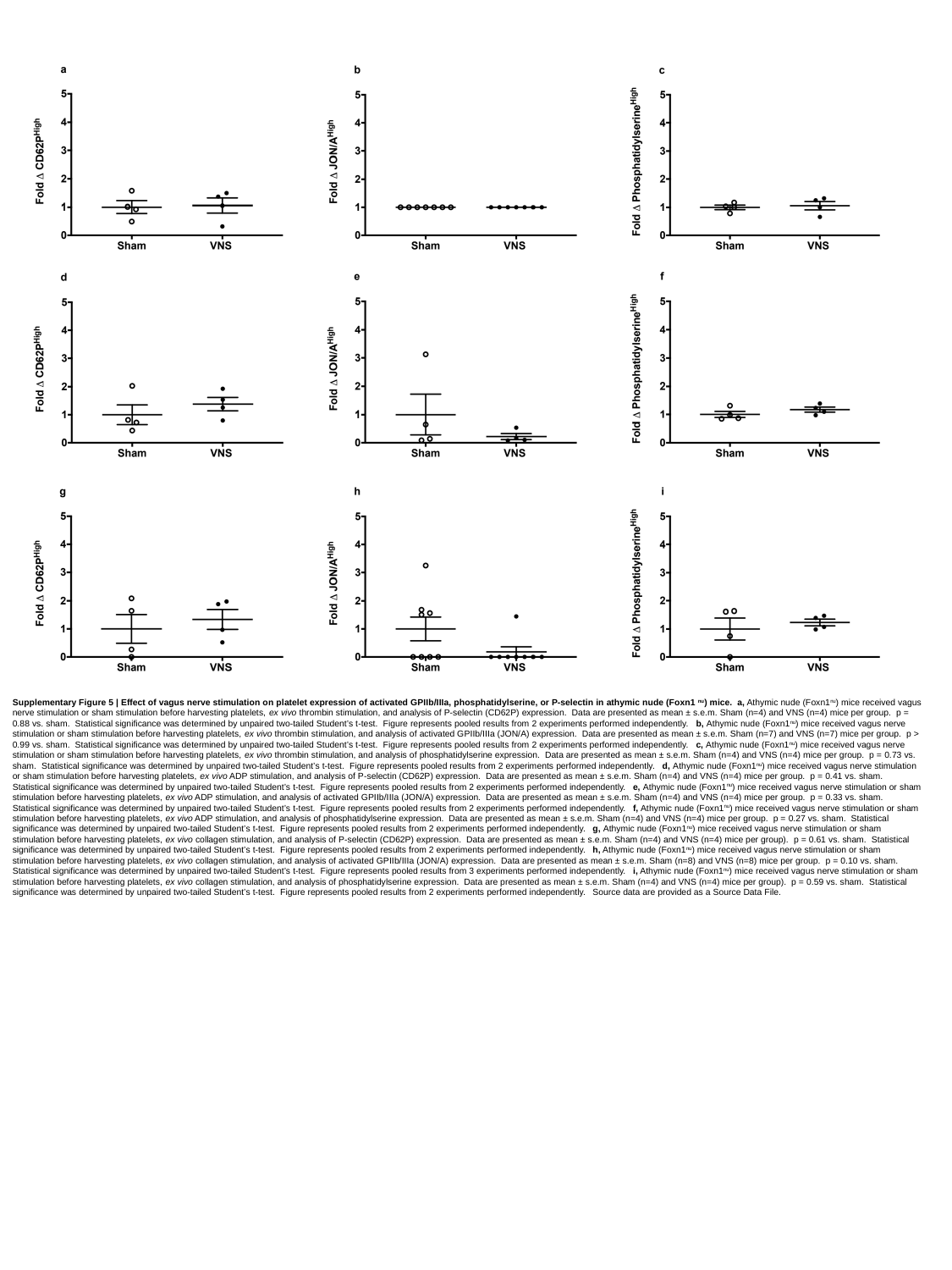

Supplementary Figure 5 | Effect of vagus nerve stimulation on platelet expression of activated GPIIb/IIIa, phosphatidylserine, or P-selectin in athymic nude (Foxn1nu) mice. a, Athymic nude (Foxn1nu) mice received vagus nerve stimulation or sham stimulation before harvesting platelets, ex vivo thrombin stimulation, and analysis of P-selectin (CD62P) expression. Data are presented as mean ± s.e.m. Sham (n=4) and VNS (n=4) mice per group. p = 0.88 vs. sham. Statistical significance was determined by unpaired two-tailed Student’s t-test. Figure represents pooled results from 2 experiments performed independently. b, Athymic nude (Foxn1nu) mice received vagus nerve stimulation or sham stimulation before harvesting platelets, ex vivo thrombin stimulation, and analysis of activated GPIIb/IIIa (JON/A) expression. Data are presented as mean ± s.e.m. Sham (n=7) and VNS (n=7) mice per group. p > 0.99 vs. sham. Statistical significance was determined by unpaired two-tailed Student’s t-test. Figure represents pooled results from 2 experiments performed independently. c, Athymic nude (Foxn1nu) mice received vagus nerve stimulation or sham stimulation before harvesting platelets, ex vivo thrombin stimulation, and analysis of phosphatidylserine expression. Data are presented as mean ± s.e.m. Sham (n=4) and VNS (n=4) mice per group. p = 0.73 vs. sham. Statistical significance was determined by unpaired two-tailed Student’s t-test. Figure represents pooled results from 2 experiments performed independently. d, Athymic nude (Foxn1nu) mice received vagus nerve stimulation or sham stimulation before harvesting platelets, ex vivo ADP stimulation, and analysis of P-selectin (CD62P) expression. Data are presented as mean ± s.e.m. Sham (n=4) and VNS (n=4) mice per group. p = 0.41 vs. sham. Statistical significance was determined by unpaired two-tailed Student’s t-test. Figure represents pooled results from 2 experiments performed independently. e, Athymic nude (Foxn1nu) mice received vagus nerve stimulation or sham stimulation before harvesting platelets, ex vivo ADP stimulation, and analysis of activated GPIIb/IIIa (JON/A) expression. Data are presented as mean ± s.e.m. Sham (n=4) and VNS (n=4) mice per group. p = 0.33 vs. sham. Statistical significance was determined by unpaired two-tailed Student’s t-test. Figure represents pooled results from 2 experiments performed independently. f, Athymic nude (Foxn1nu) mice received vagus nerve stimulation or sham stimulation before harvesting platelets, ex vivo ADP stimulation, and analysis of phosphatidylserine expression. Data are presented as mean ± s.e.m. Sham (n=4) and VNS (n=4) mice per group. p = 0.27 vs. sham. Statistical significance was determined by unpaired two-tailed Student’s t-test. Figure represents pooled results from 2 experiments performed independently. g, Athymic nude (Foxn1nu) mice received vagus nerve stimulation or sham stimulation before harvesting platelets, ex vivo collagen stimulation, and analysis of P-selectin (CD62P) expression. Data are presented as mean ± s.e.m. Sham (n=4) and VNS (n=4) mice per group). p = 0.61 vs. sham. Statistical significance was determined by unpaired two-tailed Student’s t-test. Figure represents pooled results from 2 experiments performed independently. h, Athymic nude (Foxn1nu) mice received vagus nerve stimulation or sham stimulation before harvesting platelets, ex vivo collagen stimulation, and analysis of activated GPIIb/IIIa (JON/A) expression. Data are presented as mean ± s.e.m. Sham (n=8) and VNS (n=8) mice per group. p = 0.10 vs. sham. Statistical significance was determined by unpaired two-tailed Student’s t-test. Figure represents pooled results from 3 experiments performed independently. i, Athymic nude (Foxn1nu) mice received vagus nerve stimulation or sham stimulation before harvesting platelets, ex vivo collagen stimulation, and analysis of phosphatidylserine expression. Data are presented as mean ± s.e.m. Sham (n=4) and VNS (n=4) mice per group). p = 0.59 vs. sham. Statistical significance was determined by unpaired two-tailed Student’s t-test. Figure represents pooled results from 2 experiments performed independently. Source data are provided as a Source Data File.

## Slide 6
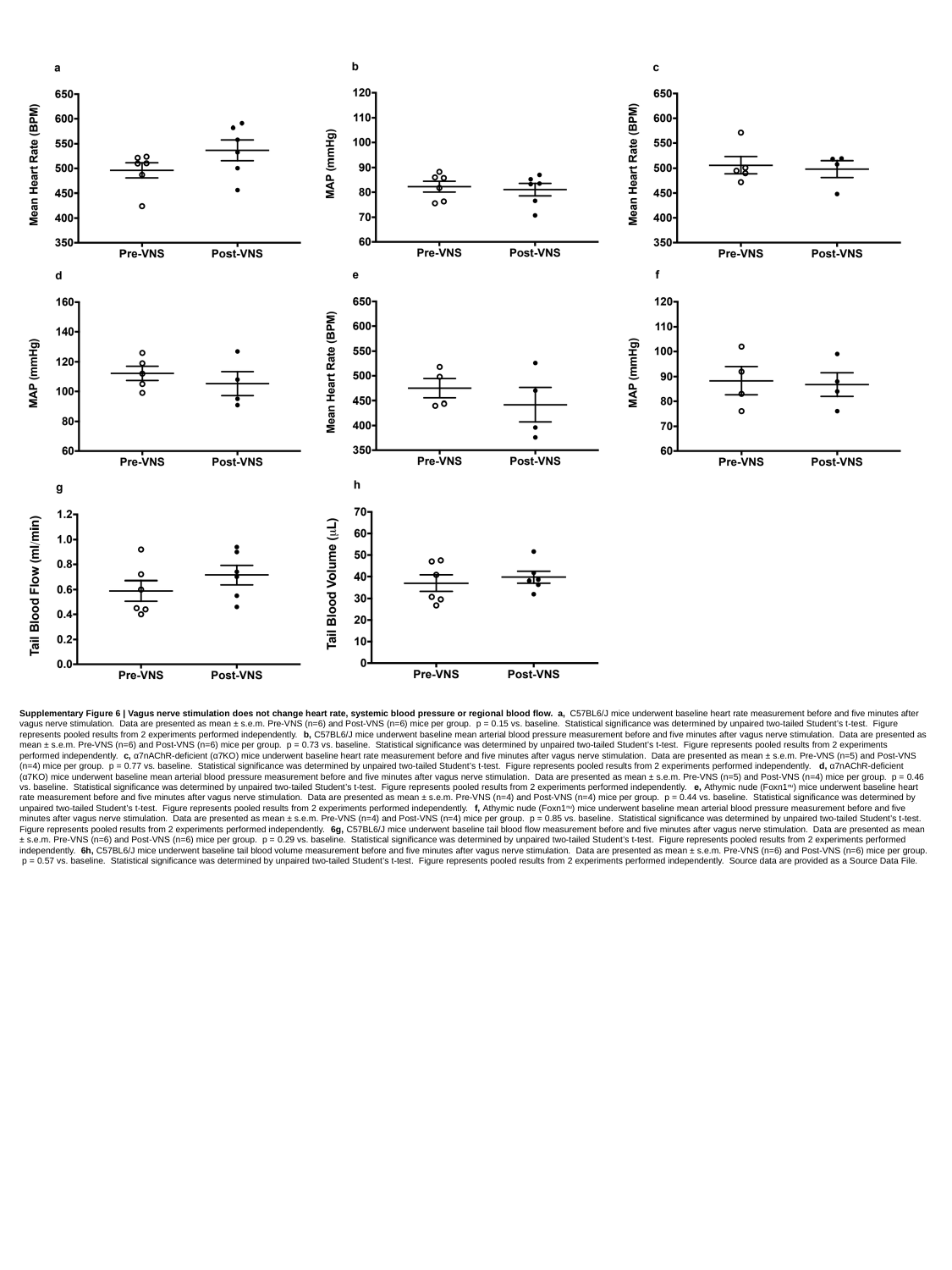

Supplementary Figure 6 | Vagus nerve stimulation does not change heart rate, systemic blood pressure or regional blood flow. a, C57BL6/J mice underwent baseline heart rate measurement before and five minutes after vagus nerve stimulation. Data are presented as mean ± s.e.m. Pre-VNS (n=6) and Post-VNS (n=6) mice per group. p = 0.15 vs. baseline. Statistical significance was determined by unpaired two-tailed Student’s t-test. Figure represents pooled results from 2 experiments performed independently. b, C57BL6/J mice underwent baseline mean arterial blood pressure measurement before and five minutes after vagus nerve stimulation. Data are presented as mean ± s.e.m. Pre-VNS (n=6) and Post-VNS (n=6) mice per group. p = 0.73 vs. baseline. Statistical significance was determined by unpaired two-tailed Student’s t-test. Figure represents pooled results from 2 experiments performed independently. c, α7nAChR-deficient (α7KO) mice underwent baseline heart rate measurement before and five minutes after vagus nerve stimulation. Data are presented as mean ± s.e.m. Pre-VNS (n=5) and Post-VNS (n=4) mice per group. p = 0.77 vs. baseline. Statistical significance was determined by unpaired two-tailed Student’s t-test. Figure represents pooled results from 2 experiments performed independently. d, α7nAChR-deficient (α7KO) mice underwent baseline mean arterial blood pressure measurement before and five minutes after vagus nerve stimulation. Data are presented as mean ± s.e.m. Pre-VNS (n=5) and Post-VNS (n=4) mice per group. p = 0.46 vs. baseline. Statistical significance was determined by unpaired two-tailed Student’s t-test. Figure represents pooled results from 2 experiments performed independently. e, Athymic nude (Foxn1nu) mice underwent baseline heart rate measurement before and five minutes after vagus nerve stimulation. Data are presented as mean ± s.e.m. Pre-VNS (n=4) and Post-VNS (n=4) mice per group. p = 0.44 vs. baseline. Statistical significance was determined by unpaired two-tailed Student’s t-test. Figure represents pooled results from 2 experiments performed independently. f, Athymic nude (Foxn1nu) mice underwent baseline mean arterial blood pressure measurement before and five minutes after vagus nerve stimulation. Data are presented as mean ± s.e.m. Pre-VNS (n=4) and Post-VNS (n=4) mice per group. p = 0.85 vs. baseline. Statistical significance was determined by unpaired two-tailed Student’s t-test. Figure represents pooled results from 2 experiments performed independently. 6g, C57BL6/J mice underwent baseline tail blood flow measurement before and five minutes after vagus nerve stimulation. Data are presented as mean ± s.e.m. Pre-VNS (n=6) and Post-VNS (n=6) mice per group. p = 0.29 vs. baseline. Statistical significance was determined by unpaired two-tailed Student’s t-test. Figure represents pooled results from 2 experiments performed independently. 6h, C57BL6/J mice underwent baseline tail blood volume measurement before and five minutes after vagus nerve stimulation. Data are presented as mean ± s.e.m. Pre-VNS (n=6) and Post-VNS (n=6) mice per group. p = 0.57 vs. baseline. Statistical significance was determined by unpaired two-tailed Student’s t-test. Figure represents pooled results from 2 experiments performed independently. Source data are provided as a Source Data File.

## Slide 7
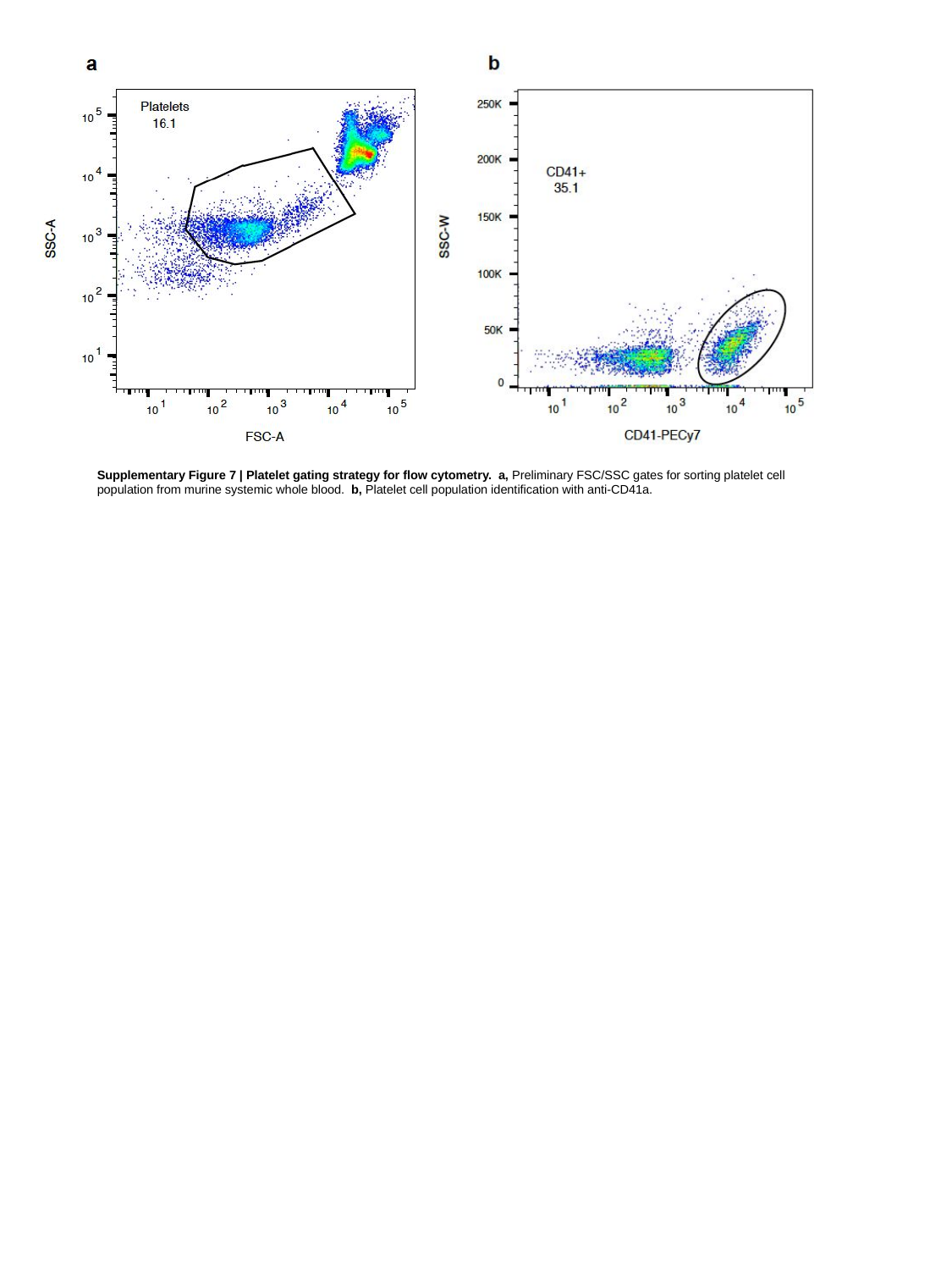

Supplementary Figure 7 | Platelet gating strategy for flow cytometry. a, Preliminary FSC/SSC gates for sorting platelet cell population from murine systemic whole blood. b, Platelet cell population identification with anti-CD41a.
