# Supplementary material for: Vagus nerve stimulation primes platelets and reduces bleeding in hemophilia A male mice
Source: Nat Commun. 2023 Jun 1;14:3122. doi: 10.1038/s41467-023-38505-6 (PMC10235098; doi:10.1038/s41467-023-38505-6)
Supplement: Supplementary file 2 — Reporting Summary [file 41467_2023_38505_MOESM2_ESM.pdf]

Corresponding author(s): Jared M. Huston
Last updated by author(s): April 30, 2023

Reporting Summary

Nature Portfolio wishes to improve the reproducibility of the work that we publish. This form provides structure for consistency and transparency in reporting. For further information on Nature Portfolio policies, see our Editorial Policies and the Editorial Policy Checklist.

Statistics

For all statistical analyses, confirm that the following items are present in the figure legend, table legend, main text, or Methods section.

n/a	Confirmed	
☐	☒	The exact sample size (*n*) for each experimental group/condition, given as a discrete number and unit of measurement
☐	☒	A statement on whether measurements were taken from distinct samples or whether the same sample was measured repeatedly
☐	☒	The statistical test(s) used AND whether they are one- or two-sided<br>*Only common tests should be described solely by name; describe more complex techniques in the Methods section.*
☒	☐	A description of all covariates tested
☐	☒	A description of any assumptions or corrections, such as tests of normality and adjustment for multiple comparisons
☐	☒	A full description of the statistical parameters including central tendency (e.g. means) or other basic estimates (e.g. regression coefficient) AND variation (e.g. standard deviation) or associated estimates of uncertainty (e.g. confidence intervals)
☒	☐	For null hypothesis testing, the test statistic (e.g. *F*, *t*, *r*) with confidence intervals, effect sizes, degrees of freedom and *P* value noted<br>*Give P values as exact values whenever suitable.*
☒	☐	For Bayesian analysis, information on the choice of priors and Markov chain Monte Carlo settings
☒	☐	For hierarchical and complex designs, identification of the appropriate level for tests and full reporting of outcomes
☒	☐	Estimates of effect sizes (e.g. Cohen's *d*, Pearson's *r*), indicating how they were calculated

Our web collection on statistics for biologists contains articles on many of the points above.

Software and code

Policy information about availability of computer code

Data collection	*Histology images were acquired with Zeiss AxioVision. IHC images were acquired with Zeiss ZEN Microsopy Software.*
Data analysis	*FlowJo v10.8.1 and BD FACSDiva Software (V9.1) were used to analyze FACS data.*

For manuscripts utilizing custom algorithms or software that are central to the research but not yet described in published literature, software must be made available to editors and reviewers. We strongly encourage code deposition in a community repository (e.g. GitHub). See the Nature Portfolio guidelines for submitting code & software for further information.

Data

Policy information about availability of data

All manuscripts must include a data availability statement. This statement should provide the following information, where applicable:

- Accession codes, unique identifiers, or web links for publicly available datasets
- A description of any restrictions on data availability
- For clinical datasets or third party data, please ensure that the statement adheres to our policy

All data supporting the findings described in this manuscript are available in the article and its Supplementary Information files. Source data are provided with this paper. There are no third party data.

Human research participants

Policy information about [studies involving human research participants and Sex and Gender in Research.](#)

Reporting on sex and gender	*Not applicable*
Population characteristics	*Not applicable*
Recruitment	*Not applicable*
Ethics oversight	*Not applicable*

Note that full information on the approval of the study protocol must also be provided in the manuscript.

Field-specific reporting

Please select the one below that is the best fit for your research. If you are not sure, read the appropriate sections before making your selection.

☒ Life sciences ☐ Behavioural & social sciences ☐ Ecological, evolutionary & environmental sciences

For a reference copy of the document with all sections, see nature.com/documents/nr-reporting-summary-flat.pdf

Life sciences study design

All studies must disclose on these points even when the disclosure is negative.

Sample size	Sample sizes were chosen based on experience with similar experimental protocols, animal strains, and outcome measures (Czura CJ et al. (2010) Shock 33, 608-613). Previously, we observed that groups of 6-8 animals with low population variance have sufficient statistical power to detect differences in treatment effect size (bleeding time) of 30-40% (alpha cut off= 0.05 and beta cut off= 0.2). We used these assumptions to determine sample sizes for our bleeding time and blood loss experiments. We also utilized these assumptions for the carotid artery injury model. For the platelet flow cytometry experiments, we hypothesized that the treatment effect size would be smaller, and the variance would be larger, which is why the sample sizes were greater.
Data exclusions	Outliers in data sets were identified using statistical software (GraphPad Prism 8). Other data exclusions occurred if there were deviations from standard protocol (i.e. technical errors), or if animals did not meet predetermined criteria for bleeding time (>20s minimum) or time to vessel occlusion (>2min minimum), as judged by at least 2 study investigators.
Replication	To verify the reproducibility of the experimental results, all experiments were carried out at least in duplicate on separate days. In addition, experimental controls were performed on each day, so cohorts were not compared to historical controls. All findings were replicated.
Randomization	Animals were randomly selected from cages and allocated into experimental groups. The investigator selecting the animals was different from the investigator determining the order of experimental manipulations (e.g. sham control vs. vagus nerve stimulation).
Blinding	For vagus nerve vs. sham stimulation experiments without other interventions, investigators were not blinded to group allocation during data collection because these functions were performed by the same individuals. For experiments involving administration of exogenous substances vs. vehicle (rFVIII, nicotine) or splenectomy vs. sham splenectomy, or administration of ChAT-eGFP+ vs. ChAT-eGFP- cells, or platelet adoptive transfer, investigators were blinded to group allocation during data collection. Outcome measures (bleeding time, clot formation) were confirmed by a minimum of 2 investigators. In most instances, investigators (minimum of 2) were not blinded to group allocation during data analysis because these functions were performed by the same individuals.

Reporting for specific materials, systems and methods

We require information from authors about some types of materials, experimental systems and methods used in many studies. Here, indicate whether each material, system or method listed is relevant to your study. If you are not sure if a list item applies to your research, read the appropriate section before selecting a response.

Materials & experimental systems

n/a	Involved in the study
☐	☒ Antibodies
☒	☐ Eukaryotic cell lines
☒	☐ Palaeontology and archaeology
☐	☒ Animals and other organisms
☒	☐ Clinical data
☒	☐ Dual use research of concern

Methods

n/a	Involved in the study
☒	☐ ChIP-seq
☐	☒ Flow cytometry
☒	☐ MRI-based neuroimaging

Antibodies

Antibodies used

JON/A, cat M023-2 IAnti-Integrin alphallbbeta3 (GPllb/llla, CD41/CD61)-PE (Emfret Analytics)
CD41, cat 133916 I Anti-CD41 Rat Monoclonal Antibody(PE (Phycoerythrin)/Cy7®) clone: MWReg30 (BioLegend)
CD62P, cat 148304 1Anti-CD62P (P-selectin) Mouse Monoclonal Antibody (APC (Allophycocyanin)) clone: RMP-1 (BioLegend)
Phosphatidylserine, cat16-256 I Anti-Phosphatidylserine Antibody, clone 1H6, Alexa Fluor" 488 (Millipore Sigma)
Alpha 7nAChR, cat ANC-007-F I Anti-Nicotinic Acetylcholine Receptor Alpha 7 (CHRNA7) (extracellular)-FITC Antibody (Alomone Labs). All antibodies above were applied 1:20 (final dilution).
Mouse CD4 Monoclonal Antibody (ThermoScientific, MA17631, 1:100), Rat Anti-CD41 antibody (Abeam, ab33661, 1:250), Donkey anti-mouse Dylight 488 (Abeam, ab96875, 1:250), Donkey anti-rat Alexa Fluor 647 (Invitrogen, #A48272, 1:250).

Validation

We provided a link for the relevant information and data sheet for each antibody. The links include the validation of all primary antibodies for the species and application, quality control procedures, and relevant citations.

JON/A- https://www.labome.com/product/Emfret-Analytics/M023-2.html
CD41- https://www.biolegend.com/en-us/global-elements/pdf-popup/pe-cyanine7-anti-mouse-cd41-antibody-7593?filename=PECyanine7 anti-mouse CD41 Antibody.pdf&pdfgen=true
CD62P- https://www.biolegend.com/en-us/global-elements/pdf-popup/apc-anti-mouse-rat-cd62p-p-selectin-antibody-10805?filename=APC anti-mouserat CD62P P-selectin Antibody.pdf&pdfgen=true
Phosphatidylserine- https://www.sigmaaldrich.com/US/en/product/mm/16256
Alpha7nAChR- https://www.alomone.com/p/anti-nicotinic-acetylcholine-receptor-%ce%b17-extracellular-fitc/ANC-007-F?b=346
Mouse CD4- https://www.thermofisher.com/antibody/product/CD4-Antibody-clone-RIV6-Monoclona1/MAl-7631
Rat Anti-CD41- https://www.abcam.com/cd41-antibody-mwreg30-ab3366l.html
Rabbit Anti-CD3- https://www.novusbio.com/products/cd3-antibody-sp7_nb600-1441
Goat anti-rabbit Alexa fluor 488- https://www.thermofisher.com/antibody/product/Goat-anti-Rabbit-lgG-Heavy-chain-Secondary-Antibody-Recombinant-Polyclonal/A27034
Donkey anti-mouse Dylight 488- https://www.abcam.com/donkey-mouse-igg-hl-dylight-488-ab96875.html
Donkey anti-rat Alexa Fluor 647- https://www.thermofisher.com/antibody/product/Donkey-anti-Rat-lgG-H-L-Highly-Cross-Adsorbed-Secondary-Antibody-Polyclonal/A48272

Anti-JON/A: Pircher J, Czermak T, Ehrlich A, Eberle C, Gaitzsch E, Margraf A, et al. Cathelicidins prime platelets to mediate arterial thrombosis and tissue inflammation. Nat Commun. 2018;9: 1523

Anti-CD41: Palpant NJ, Pabon L, Rabinowitz JS, Hadland BK, Stoick-Cooper CL, Paige SL, Bernstein ID, Moon RT, Murry CE. Transmembrane protein 88: a Wnt regulatory protein that specifies cardiomyocyte development. Development. 2013 Sep;140(18): 3799-808.
-Fujii T, Sakata A, Nishimura S, Eto K, Nagata S. TMEM16F is required for phosphatidylserine exposure and microparticle release in activated mouse platelets. Proc Natl Acad Sci US A. 2015 Oct 13;112(41): 12800-5.

Anti-CD62P: Gao Y, Sarode A, Kokoroskos N, Ukidve A, Zhao Z, Guo S, Flaumenhaft R, Gupta AS, Saillant N, Mitragotri S. A polymer-based systemic hemostatic agent. Sci Adv. 2020 Jul 31;6(31): eaba0588.

Anti-Phosphatidylserine: Weir MC, Hellwig S, Tan L, Liu Y, Gray NS, Smithgall TE. Dual inhibition of Fes and Flt3 tyrosine kinases potently inhibits Flt3-ITD+ AML cell growth. PLoS One. 2017 Jul 20;12(7): e0181178.
-Mandinov L, Mandinova A, Kyurkchiev S, Kyurkchiev D, Kehayov I, Kolev V, Soldi R, Bagala C, de Muinck ED, Lindner V, Post MJ, Simons M, Bellum S, Prudovsky I, Maciag T. Copper chelation represses the vascular response to injury. Proc Natl Acad Sci US A. 2003 May 27;100(11): 6700-5.
-Lucchetti D, Battaglia A, Ricciardi-Tenore C, Colella F, Perelli L, De Maria R, Scambia G, Sgambato A, Fattorossi A. Measuring Extracellular Vesicles by Conventional Flow Cytometry: Dream or Reality? Int J Mol Sci. 2020 Aug 29;21(17): 6257.

Anti-Alpha7nAChR: Haberberger RV, Bernardini N, Kress M, Hartmann P, Lips KS, Kummer W. Nicotinic acetylcholine receptor subtypes in nociceptive dorsal root ganglion neurons of the adult rat. Auton Neurosci. 2004;113(1-2): 32-42.

Animals and other research organisms

Policy information about studies involving animals; ARRIVE guidelines recommended for reporting animal research, and Sex and Gender in Research

Laboratory animals

Adult male 8-12 week old BALB/c mice (20-25 g, Taconic), adult male 8-12 week old C57BL6/J mice (20-25 g, Jackson Labs), adult male 8-16 week old α7nAChR-deficient mice (20-25 g, Jackson Labs, C57BL6/J background) and wild-type littermates, adult male 8-12 week old (Foxn1nu) nude mice (20-25 g Taconic, BALB/c background), adult male 8-12 week old factor VIII knockout mice (20-25 g, Jackson Labs), adult male 8-12 week old ChAT(BAC)-EGFP mice (20-25 g, Jackson Labs), and adult male 8-12 week old ChAT-TdTomato mice generated from crossing ChAT-Cre mice (B6;129S6-Chattm2(cre)Lowl/J, Jackson Labs #006410) with Td-Tomato mice (B6.Cg-Gt(ROSA)26Sortm14(CAG-tdTomato)Hze/J, Jackson Labs #007914) expressing red fluorescent protein in cholinergic cells are used for experiments. All animals are housed at 22°C (range 20-26°C) and 42% humidity (range 30-70%) on a 12-hour light/dark cycle. Foxn1nu nude mice are housed in a room devoted to immunocompromised animals. Standard animal chow and water are freely available. Food and water are not withheld before experiments. Following survival surgery, animals are provided a warming blanket and administered 0.9% normal saline (30-50 mL/kg, s.c.). Animals return to normal housing after achieving sternal recumbency and ambulation. Animals are monitored twice daily for 72 hours post-operatively and given analgesia (Buprenex 0.1 mL/kg, s.c.) at each visit. After 72 hours, animals are monitored twice weekly until termination of the experiment. Humane endpoints for early euthanasia include inability to stand, agonal, rapid or labored breathing, significant decreases in activity score, sick posturing, decreased grooming or reduced response to touch. Animals are euthanized by gradual displacement CO2 asphyxiation and terminal exsanguination under anesthesia. Endpoints for early anesthesia for bleeding experiments include lethal blood loss, increased pallor, drying of mucous membranes, or writhing.

Wild animals

No wild animals were used in the study.

Reporting on sex

Findings apply to only male sex.

Field-collected samples

No field-collected samples were used in the study.

Ethics oversight

All animal experiments are performed in accordance with the National Institutes of Health (NIH) Guidelines under protocols approved by the Institutional Animal Care and Use Committee of The Feinstein Institutes for Medical Research.

Note that full information on the approval of the study protocol must also be provided in the manuscript.

Flow Cytometry

Plots

Confirm that:

- [x] The axis labels state the marker and fluorochrome used (e.g. CD4-FITC).
- [x] The axis scales are clearly visible. Include numbers along axes only for bottom left plot of group (a 'group' is an analysis of identical markers).
- [x] All plots are contour plots with outliers or pseudocolor plots.
- [x] A numerical value for number of cells or percentage (with statistics) is provided.

Methodology

Sample preparation

As described in the Methods section, systemic blood from the inferior vena cava is collected 17 minutes after vagus nerve stimulation (5 minutes) into Tris buffered saline (20mM Tris-HCl, 137mM NaCl, pH 7.3) containing 20U/mL heparin. Heparinized whole blood is then diluted with modified Tyrode's Buffer (134 mM NaCl, 0.34 mM Na2HPO4, 2.9 mM KCl, 12 mM NaHCO3 20 mM Hepes, pH 7.0 with 5mM glucose, 0.35% BSA), stimulated ex vivo with thrombin (1U/ml), collagen (0.5 or 5 ˜g/ml), or ADP (20 ˜g/ml), and stained with antibodies against CD41a (BioLegend), CD62P (P-Selectin) (BioLegend), active GPIIb/IIIa (JON/A) (Emfret Analytics), and phosphatidylserine (Millipore Sigma). In separate experiments, non-stimulated platelets are stained with anti-nicotinic acetylcholine receptor alpha7 (CHRNA7) (extracellular)-FITC antibody (Alomone Labs).

Instrument

BD FACSymphony A3 Cell Analyzer with Blue (488nm), Violet (405nm), Red (630nm), Yellow-Green (564 nm) and UV (325 nm) lasers.
BD LSR Fortessa with Blue (488nm), Red (633nm), Green (532nm), and Violet (405nm) lasers.

Software

BD FACSDiva Software V9.1 or later for data collection.
FlowJo 10.8.1 was used for data analysis.

Cell population abundance

Platelets were not enriched or sorted prior to ex vivo stimulation. Platelet abundance ranged from 10-24% relative to all events (30K events/sample) collected from murine whole blood.

Gating strategy

The preliminary gating strategies are shown in Supplementary Fig. 7a, 7b. The starting platelet cell population from murine whole blood was identified by forward and side scatter plots (FSC-A/SSC-A) followed by staining with anti-CD41a (BioLegend).

- [x] Tick this box to confirm that a figure exemplifying the gating strategy is provided in the Supplementary Information.
